# Physic-informed deep operator networks for modeling 2D time-domain electromagnetic wave propagation in various media

**DOI:** 10.1016/j.isci.2026.115076

**Published:** 2026-02-18

**Authors:** Sooyoung Oh, EungKyu Lee, Sun K. Hong

**Affiliations:** 1 School of Electronic Engineering, Soongsil University, Sangdo-ro 369, Seoul 06978, South Korea; 2 Department of Electronic Engineering, Kyung Hee University, Deogyeong-daero, Giheung-gu, Yongin-si, Gyeonggi-do 17104, South Korea; 3 Department of Intelligent Semiconductor, Soongsil University, Sangdo-ro 369, Seoul 06978, South Korea

**Keywords:** Physics, Applied sciences

## Abstract

Accurate prediction of time-domain electromagnetic (EM) waves is essential for designing high-frequency systems in complex environments. Traditional finite-difference time-domain (FDTD) solvers become burdensome when handling a large domain, motivating alternative approaches. Physics-informed neural networks (PINNs) offer data-efficient frameworks by embedding physical constraints, but their generalization ability is limited, often requiring retraining when source locations or material configurations change. In this work, we investigate a physics-informed deep operator network (PI-DeepONet), for modeling two-dimensional transient EM wave propagation by incorporating the time-domain Helmholtz equation. Leveraging the operator-learning structure of DeepONet, the proposed framework demonstrates enhanced generalization across diverse excitation and material conditions, including multi-source in free-space and inhomogeneous media. Each trained model predicts wave propagation and scattering for arbitrary source and scatterer configurations, including movable dielectric inclusions. The predicted spatiotemporal fields are quantitatively compared with FDTD simulations to validate accuracy and assess the model’s potential as an efficient surrogate for time-domain EM analysis.

## Highlights

• PI-DeepONet learns a physics-informed operator for 2D transient EM waves
• One trained model generalizes across source and dielectric inclusion locations
• Matches FDTD with low RMSE and up to 15 times faster full-field prediction
• Energy analysis confirmeds bounded, physically consistent time evolution

## Graphical abstract

**Figure:**
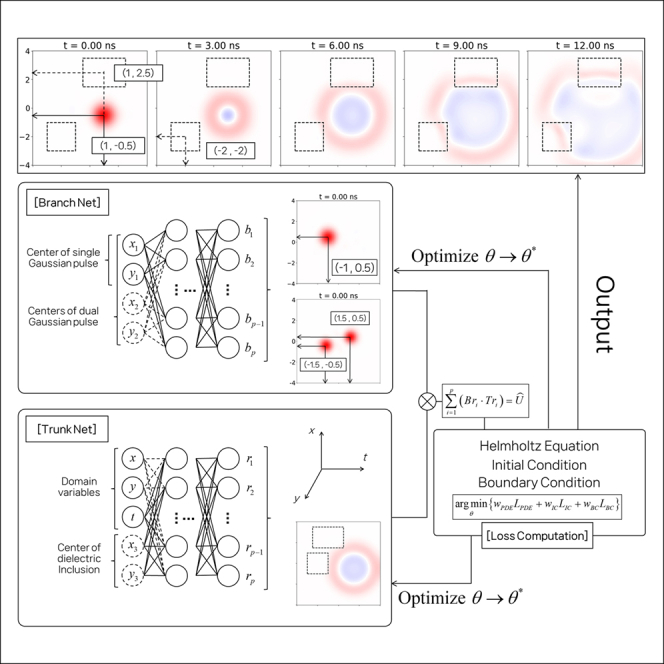

## Introduction

Accurate prediction of the spatiotemporal behavior of electromagnetic (EM) waves is a prerequisite for the design, analysis, and optimization of high-frequency EM systems. The demand for precise EM modeling and analysis has grown across a wide range of applications including advanced antenna design,^1^ radar-based non-destructive evaluation (NDE),^2^^,^^3^ ground-penetrating radars (GPRs),^4^^,^^5^ and wave propagation/focusing in complex environments.^6^^,^^7^ These scenarios often feature spatially inhomogeneous media, irregular boundaries, and multiple wave sources, leading to occurrences of physical phenomena such as wave interference, reflection, and refraction. As a result, accurately modeling such interactions is a key aspect in EM analysis.

Traditional numerical methods – most notably the finite-difference time-domain (FDTD) scheme – have long served as the cornerstone for modeling transient EM wave behavior, offering time-resolved solutions to Maxwell’s equations across discretized space-time grids. Owing to their simplicity and robustness, FDTD methods are widely used in simulating wave propagation, scattering, and field interactions in various EM systems. However, despite their well-established utility, FDTD often faces difficulties when applied to large simulation domains with fine geometric details. For instance, when simulating wave propagation, scattering, and focusing in large-scale environments such as indoor spaces, FDTD requires the entire domain to be discretized with fine spatial and temporal grids, resulting in a significant increases the computational burden. Additionally, FDTD is subject to stability constraints that limit the maximum time step. Moreover, FDTD must be recomputed from scratch for each new source or material configuration, posing challenges for parameter sweeps, inverse design, or real-time applications. Consequently, although FDTD-based solvers remain highly accurate and widely trusted, their application to electrically large and complex domains often demands substantial computational resources and long simulation times, which can be a limiting factor in iterative design or real-time analysis scenarios. These limitations have motivated the development of alternative modeling approaches that are more flexible and computationally scalable, particularly for complex or large-scale EM environments.

Since the introduction of physics-informed neural networks (PINNs) by Raissi et al.^8^ 2019, they have rapidly evolved as a promising framework that integrates governing physical laws directly into the training of neural networks. Unlike traditional purely data-driven models, PINNs embed partial differential equations (PDEs), boundary conditions (BCs), and initial conditions (ICs) into the loss function itself, allowing the network to learn physically constrained solutions without requiring large labeled datasets. Specifically, during training, the neural network takes space-time coordinates as inputs and outputs the corresponding target physical variables; the gradients of these outputs with respect to inputs are computed via automatic differentiation and used to evaluate the residuals of underlying PDEs. These residuals, along with any supervised loss from available measurements or known conditions, are minimized simultaneously – resulting in a data-free, physics-constrained surrogate model. This paradigm enables PINNs to solve a wide range of physics-driven problems in a data-efficient manner, particularly in domains where obtaining high-fidelity labeled data is expensive or impractical. Applications span across various areas, including thermal analysis,^9^ fluid mechanics,^10^^,^^11^ and wave dynamics,^12^ as well as kinetic transport phenomena.^13^^,^^14^ Among the various domains where PINNs have been applied, EM problems have emerged as a particularly active area of research, prompting a series of studies focusing on solving Maxwell’s equations using this framework.

Zhang et al.^15^ proposed a PINN framework for solving time-domain Maxwell’s equations, demonstrating its applicability to one-dimensional (1D) and two-dimensional (2D) scenarios. An inverse approach was considered by Zhang et al.,^16^ where the reconstruction of inhomogeneous plasma parameters in 1D magnetized environments is enabled using sparse sampling. Further expanding on inverse design applications, Liu^17^ introduced knowledge-embedded PINNs to optimize horn antenna structures, effectively addressing 2D radiation patterns, but within a static design configuration. The domain-adaptive PINN proposed by Piao et al.^18^ demonstrated effective handling of 2D heterogeneous media with irregular interfaces, yet its focus was limited to parameter estimation rather than comprehensive time-domain field evolution. Moreover, a time-domain PINN-based MaxwellNet model that handles both forward and inverse problems with varying material distributions was proposed by Liu et al.^19^ More recently, PINN architectures have also been tailored to challenging electromagnetic media; for example, multi-receptive-field PINNs have been reported for nano-optical scattering in complex dispersive/anisotropic/nonlinear/chiral materials with near real-time field reconstruction.^20^ In parallel, physics-augmented surrogate Maxwell solvers such as WaveY-Net combine data-driven learning with discrete Maxwell constraints to enable ultrafast field prediction and to accelerate gradient-based freeform nanophotonic optimization.^21^

Beyond Maxwell-specific formulations, PINN research has rapidly progressed in broader wave-physics settings, where recent studies have demonstrated improved training stability and accuracy through methodological enhancements such as decomposition and modified training strategies. For instance, multi-domain/domain-decomposition PINN variants have been used to capture complex nonlinear wave dynamics and to support parameter discovery in nonlinear Schrödinger-type systems.^22^ Likewise, mix-training strategies that incorporate additional residual terms and revised training regions have been reported to improve the reconstruction of strongly nonlinear/nonlocal wave solutions associated with inverse problems.^23^^,^^24^ Moreover, time-domain decomposition PINNs have been introduced to mitigate long-time integration difficulty by splitting the temporal domain and enforcing consistency across sub-interval interfaces.^25^ These works highlight the fast-evolving PINN methodology and motivate a continued development of an architecture that can be applied to general wave-physics problems.

However, many practical EM scenarios are inherently dynamic, with source locations or material inclusions subject to variation over time or configuration changes. Modeling such dynamic scenarios introduces significant challenges due to increased complexity in wave interactions. Despite the importance of capturing these phenomena, there has been little progress in developing PINN architectures capable of handling such configurations. Furthermore, limitations of conventional PINNs lie in their unknown generalization capability, as they are typically trained as instance-specific solvers that should be re-trained from scratch for each new configuration of source location or environmental condition. One strategy to improve generalization is to explicitly parameterize the input by augmenting the network with additional inputs that describe system variations (e.g., source location and material properties). However, this approach often increases the complexity of the optimization landscape and may lead to degraded accuracy or convergence issues. Consequently, the inability to generalize across source and inclusion positions makes it difficult to obtain rapid and reliable solutions, rendering many real-world applications computationally prohibitive for conventional PINNs or FDTD solvers.

A promising route to enhance the generalization capability of physics-informed modeling is operator learning, which aims to learn the mapping (operator) from problem inputs – such as source/initial-condition descriptors and material/geometry parameters – to the corresponding solution fields across a family of configurations. Among neural-operator architectures, two representative approaches are the Fourier Neural Operator (FNO)^26^^,^^27^^,^^28^ and deep operator networks (DeepONets).^29^^,^^30^^,^^31^ FNO learns solution operators efficiently on structured grids using global spectral convolutions, and it has demonstrated strong performance for many parametric PDE problems when abundant grid-based training data are available. By contrast, DeepONets represent operators through a branch-trunk decomposition that naturally supports conditioning on parametric inputs and pointwise evaluation at arbitrary space-time coordinates, which is particularly convenient for transient wave problems where collocation-based physics enforcement and flexible sampling are desirable. Qi et al.^32^ demonstrated the applicability of the physics-informed DeepONet (PI-DeepONet) framework to three-dimensional time-domain EM modeling. Their results showed that a single trained model could accurately predict field distributions across different microwave circuits and metasurface configurations, achieving errors on the order of 10^−3^ while maintaining computational efficiency comparable to or faster than conventional FDTD solvers. Moreover, the framework exhibited strong generalizability, successfully handling geometric and material uncertainties without the need for retraining, thereby validating the potential of operator-based learning for large-scale electromagnetic problems. Wang^33^ proposed a transformer-based operator-learning “multiphysics field solver” that hybridizes deep learning with classical FEM: the network takes coarse-grid FEM results as input and predicts the corresponding dense-grid field solution, while using a loss that incorporates physical constraints. After training, the framework enables quasi-real-time field prediction on GPUs, and even when the coarse-grid FEM preprocessing step is included, the overall runtime remains substantially lower than running dense-grid FEM alone.

Building upon this advancement, this article presents and validates a PI-DeepONet framework specifically developed for modeling two-dimensional (2D) transient EM wave propagation. Unlike previous studies focusing on static structural analysis, the present work emphasizes the accurate prediction of dynamic wave propagation behaviors across space and time. By incorporating the time-domain Helmholtz equation as the governing PDE and training the model to learn a parametric solution operator that maps finite-dimensional, source-dependent excitation descriptors (e.g., the centers of single/dual Gaussian pulses) together with material/geometry parameters to spatiotemporal field solutions, the proposed framework captures complex field interactions – such as wave interference, reflection, and superposition – even in environments containing dielectric inclusions. Unlike coordinate-to-field PINNs that regress the field directly from (*x*, *y*, *t*) and parameters, our PI-DeepONet learns a descriptor-conditioned solution operator via a branch-trunk decomposition, enabling efficient reuse of trunk features on fixed spatiotemporal grids. Furthermore, a single trained PI-DeepONet model can generalize across varying source locations and dielectric inclusion positions, enabling accurate predictions of spatiotemporal field evolution without retraining. In addition, the network is trained under a variety of environmental settings and boundary conditions to enhance its robustness and adaptability across diverse propagation scenarios. The generalization capability is rigorously evaluated under various excitation setups, including single and dual Gaussian sources, and the predicted results are quantitatively compared with high-fidelity FDTD simulations, demonstrating strong agreement and validating the accuracy and applicability of the proposed approach.

## Results

### Network configuration of physics-informed deep operator network

**Figure 1:**
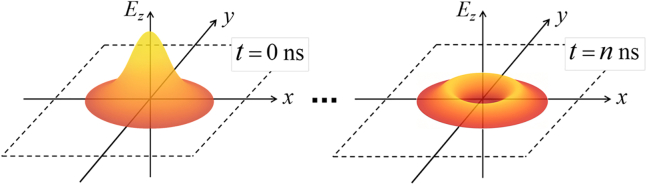
Spatial coordinate configuration and electric field orientation of 2D TM_z_

In this study, we consider a 2D transverse magnetic (TM_z_) configuration, where the behavior of the EM wave can be characterized by a scalar electric field component, *U* (representing *E*_*z*_), governed by the time-domain Helmholtz equation as:

(Equation 1) $$\frac{\partial^{2}U}{\partial t^{2}}={\left(\frac{1}{\sqrt{\varepsilon_{0}\varepsilon_{r}\left(x,y\right)\mu_{0}\mu_{r}\left(x,y\right)}}\right)}^{2}\left(\frac{\partial^{2}U}{\partial x^{2}}+\frac{\partial^{2}U}{\partial y^{2}}\right)\text{,}$$

where *ε*_0_ is the vacuum permittivity, *μ*_0_ is the vacuum permeability, and *ε*_*r*_(*x*,*y*), *μ*_*r*_(*x*,*y*) are the spatially varying relative permittivity and relative permeability of the medium, respectively. The coordinate configuration and orientation are illustrated in Figure 1. Building upon this setup, we construct a PI-DeepONet framework capable of handling a diverse set of EM environments. Specifically, four distinct scenarios are considered: 1) free-space with a single Gaussian source, 2) free-space with dual Gaussian sources, 3) a dielectric-inclusion medium with a single Gaussian source, 4) multiple dielectric-inclusion media with a single Gaussian source. In all cases, the spatial locations of the sources are not fixed but treated as variable inputs during training, enabling the model to generalize across a wide range of excitation configurations. In the dielectric-inclusion cases, not only the source positions but also the position of the dielectric inclusion is parameterized–allowing the model to accurately capture complex wave interactions arising from arbitrary source-scatterer arrangements. Moreover, to simulate diverse propagation scenarios of EM waves, both reflecting and absorbing boundary conditions are implemented, enabling the framework to effectively model wave behaviors under different environmental settings.

**Figure 2:**
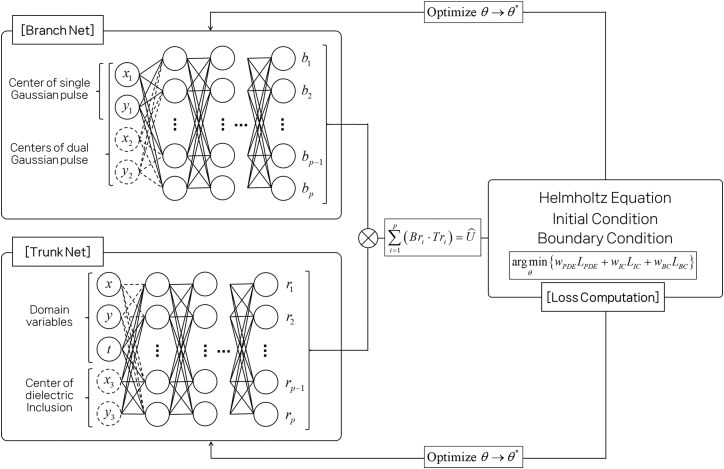
Network configuration of PI-DeepONet to model 2D EM wave propagation

Figure 2 shows the network configuration of the proposed PI-DeepONet framework. The architecture is composed of two neural subnetworks: the Branch Net and the Trunk Net. The Branch Net receives input representations associated with the excitation source configuration, i.e., parameters that describe the center location of the Gaussian pulses. The Trunk Net, on the other hand, takes the coordinates of the evaluation domain as input. In the simplest case of free-space with a single Gaussian source, the domain variable (*x*, *y*, *t*) alone is used as input to the Trunk Net, and only the solid-line input paths are activated.

When extending to more complex scenarios involving multiple sources or a dielectric-inclusion medium, the input space is augmented accordingly. For instance, in the dual-source case, the Branch Net is supplied with the parameters corresponding to both Gaussian pulses, while the Trunk Net receives the same domain coordinates. In cases involving dielectric inclusion, the Trunk Net additionally takes the spatial location of the dielectric block as a part of its input, allowing the network to capture the influence of varying material distributions on wave propagation. These additional variables, shown as dashed lines in Figure 2, can be flexibly added depending on the complexity of the simulation scenario, enabling a single trained model to generalize across a family of problems with different source and material configurations. This modular input design allows the PI-DeepONet to learn a generalized mapping from source functions and domain configurations to the corresponding spatiotemporal field solutions. Note that, while this modular input design supports generalization over continuously varying source/material descriptors within a scenario, cases with different input parameterizations are handled by training separate models tailored to each scenario.

To ensure a balanced model capacity across different configurations, both the Branch and Trunk networks are implemented with the same architectural specifications–identical depth, width, activation functions, and output dimensions–although they process different types of input tensors. The first two columns in Table 1 show the detailed network specifications used for each scenario. The number of neurons and the depth of the hidden layers are adjusted according to the complexity of the configuration. For all cases, the activation function used in both subnetworks is the hyperbolic tangent (tanh) function. Its smooth and differentiable nature allows for effective gradient flow during training and provides the ability to capture both positive and negative field variations–an essential characteristic for modeling physics-based problems involving wave phenomena.

**Table 1:** PI-DeepONet network and training configurations across four representative environments

Configuration		# of Neurons	# of Layers	Epoch	N_PDE_	N_IC_	N_BC_	Training Time	GPU Memory footprint
Case 1^a^	ABC	64	20	100,000	50,000	10,000	10,000	366 min	16.43 GB
Case 2^b^	ABC	128	30	100,000	50,000	30,000	10,000	382 min	32.43 GB
	RBC	128	30	100,000	50,000	30,000	10,000	375min	32.43 GB
Case 3^c^	ABC	128	30	150,000	70,000	30,000	10,000	552min	32.43 GB
	RBC	128	30	150,000	70,000	30,000	10,000	548min	32.43 GB
Case 4^d^	ABC	128	30	150,000	50,000	50,000	10,000	567min	32.43 GB
	RBC	128	30	150,000	50,000	50,000	10,000	560min	32.43 GB

a Free-space/single-source.

b Free-space/dual-source.

c Dielectric-inclusion/single-source.

d Multi-inclusion/single-source.

### Loss configuration

As mentioned above, the network is trained to predict the scalar electric field component *U*(*x*,*y*,*t*) governed by the time-domain Helmholtz equation, as shown in Figure 2. Unlike standard PINNs, where the neural network directly maps spatiotemporal inputs to predicted field values, the final output $\hat{U}\left(x,y,t\right)$ of PI-DeepONet is computed as the inner product between the outputs of two subnetworks as

(Equation 2) $$\hat{U}\left(x,y,t\right)=\sum_{i=1}^{p}Br_{i}\cdot Tr_{i}\text{,}$$

where *Br*_*i*_ and *Tr*_*i*_ are the output components of the Branch and Trunk networks, respectively, and *p* is the dimensionality of the latent feature space. In the formulation, the Branch Net takes the configuration descriptors (e.g., the Gaussian source-center coordinates and additional source parameters in the multi-source cases) and encodes them into a *p-*dimensional latent vector. This latent vector can be interpreted as scenario-dependent coefficients that summarize how the particular excitation activates different underlying field patterns. The Trunk Net, on the other hand, takes the spatiotemporal query coordinates (and, when applicable, inclusion descriptors such as the inclusion center location) and produces another *p*-dimensional latent vector that acts as a set of shared spatiotemporal features evaluated at that point. The final prediction is obtained by combining these two latent vectors so that the field at (*x*, *y*, *t*) is represented as a weighted mixture of trunk features, with weights determined by the branch embedding. For inclusion problems, we additionally condition the Trunk Net on inclusion descriptors because the inclusion geometry can influence the field globally through scattering and local wave-speed contrast. Supplying these descriptors enables the Trunk Net to produce location-aware spatiotemporal features that more accurately capture interface-driven pattern changes, while the Branch Net continues to encode the excitation parameters. Overall, (2) can be viewed as forming the predicted field by combining configuration-dependent coefficients (from the Branch Net) with coordinate-dependent latent features (from the Trunk Net), resulting in a parametric surrogate that generalizes across transient EM scenarios indexed by the chosen excitation and medium parameters.

The total loss function used to train PI-DeepONet is constructed in a physics-informed manner, incorporating the governing equation and its associated constraints. It consists of three primary components:

(Equation 3) $$L_{total}=w_{PDE}L_{PDE}+w_{IC}L_{IC}+w_{BC}L_{BC}\text{.}$$

where *L*_*PDE*_, *L*_*IC*_, and *L*_*BC*_ refer to the PDE, initial condition, and boundary condition loss, respectively, and *w*_*PDE*_, *w*_*IC*_, and *w*_*BC*_ represent the corresponding weights. The first term, *L*_*PDE*_, penalizes the residual of the time-domain Helmholtz equation, evaluated at *N*_*PDE*_ points in the computational domain as

(Equation 4) $$L_{PDE}=\frac{1}{N_{PDE}}\sum_{i=1}^{N_{PDE}}{\left[\frac{\partial^{2}{\hat{U}}_{i}}{\partial t^{2}}-{\left(\frac{c}{\sqrt{\varepsilon_{r}\left(x_{i},y_{i}\right)\mu_{r}\left(x_{i},y_{i}\right)}}\right)}^{2}\left(\frac{\partial^{2}{\hat{U}}_{i}}{\partial x^{2}}+\frac{\partial^{2}{\hat{U}}_{i}}{\partial y^{2}}\right)\right]}^{2}\text{,}$$

where ${\hat{U}}_{i}$ denotes the predicted value (${\hat{U}}_{i}$ equals to ${\hat{U}}_{i}\left(x,y,t\right)$) at the *i*^*th*^ collocation point, and *ε*_*r*_(*x*_*i*_,*y*_*i*_), *μ*_*r*_(*x*_*i*_,*y*_*i*_) are the relative permittivity and permeability distributions at the *i*^*th*^ collocation point, respectively. The *N*_*PDE*_ sample points are randomly distributed across the entire spatial and temporal domain to evaluate the residual of the governing equation. A larger number of collocation points is used in inhomogeneous media cases to capture the more complex field variations arising from material discontinuities, as shown in Table 1.

To initiate the wave propagation without explicitly introducing an external source term in the governing equation, a Gaussian pulse is imposed as the initial field distribution *U*(*x*,*y*,0). The localized initial condition acts as a transient perturbation that initiates wave propagation, allowing the network to learn field dynamics solely based on the PDE and initial/boundary constraints.^12^ The initial condition loss (*L*_*IC*_) is defined as

(Equation 5) $$L_{IC}=\frac{1}{N_{IC}}\sum_{i=1}^{N_{IC}}{\left[\hat{U}\left(x,y,0\right)-U\left(x,y,0\right)\right]}^{2}\text{,}$$

where $\hat{U}\left(x,y,0\right)$ is the predicted initial value, *N*_*IC*_ is the number of sample points of initial time. For *N*_*IC*_, they are sampled from the spatial domain at *t* = 0, representing the moment when the Gaussian pulse is initially imposed. In the dual-source configuration and dielectric inclusion cases, a larger number of initial condition points are utilized than in the single-source excitation.

For boundary conditions, both reflecting and absorbing boundary conditions are considered to emulate diverse propagation scenarios. For the reflecting case, Neumann boundary conditions (i.e., ∂*U*/∂*x* = 0, ∂*U*/∂*y* = 0) are applied on all edges of the spatial domain. These are incorporated into the loss function as

(Equation 6) $$L_{BC}=\frac{1}{N_{BC}}{\sum_{i=1}^{N_{BC}}\left[\frac{\partial {\hat{U}}_{i}}{\partial x}\right]}^{2}+\frac{1}{N_{BC}}{\sum_{i=1}^{N_{BC}}\left[\frac{\partial {\hat{U}}_{i}}{\partial y}\right]}^{2}\text{,}$$

where *N*_*BC*_ represents the number of sample points at each axis boundary. These points are independently sampled along *x*∈*L*_*x*_/2 and *y*∈*L*_*x*_/2 boundaries to ensure that the normal derivatives are sufficiently enforced. In addition, an absorbing boundary condition (ABC) is imposed on the outer boundary to emulate open-space radiation. Instead of using a global constant wave velocity, we treat the phase velocity as a local quantity evaluated at each boundary collocation point (*x*_*i*_,*y*_*i*_). For the non-magnetic dielectric inclusion considered in this work, the local wave velocity is computed as $c\left(x,y\right)=c_{0}/\sqrt{\varepsilon_{r}\left(x,y\right)}$. This local velocity is incorporated into the first-order approximation of the form

(Equation 7) $$\frac{\partial {\hat{U}}_{i}}{\partial t}+c\left(x,y\right)\frac{\partial {\hat{U}}_{i}}{\partial n}=0\text{,}$$

where ∂/∂*n* denotes the normal derivative along the boundary. The loss term for the ABC is then defined as the residual of the above equation at each boundary, expressed as

(Equation 8) $$L_{BC}=\frac{1}{N_{BC}}\sum_{i=1}^{N_{BC}}\left[{\left(\frac{\partial {\hat{U}}_{i}}{\partial t}+c_{i}n_{x}\frac{\partial {\hat{U}}_{i}}{\partial x}\right)}^{2}+{\left(\frac{\partial {\hat{U}}_{i}}{\partial t}+c_{i}n_{y}\frac{\partial {\hat{U}}_{i}}{\partial y}\right)}^{2}\right]\text{,}$$

where *n*_*x*_,*n*_*y*_∈[-1 or 1] indicate the direction of the outward normal for the x and y boundaries, respectively, and *c*_*i*_≡*c*(*x*_*i*_,*y*_*i*_) is the local phase velocity evaluated at each boundary point. These additional terms allow the network to effectively absorb outgoing waves and emulate open-space propagation conditions without reflection. Each weight value utilized in each configuration is reported in Table 2.

**Table 2:** Weights in the loss functions used for each configuration

Configuration		w_PDE_	w_IC_	w_BC_
Case 1^a^	ABC	2.5	5.0	1.0
Case 2^b^	ABC	2.5	5.0	1.0
	RBC	2.5	5.0	1.0
Case 3^c^	ABC	1.0	5.0	1.0
	RBC	5.0	5.0	1.0
Case 4^d^	ABC	2.5	5.0	1.0
	RBC	5.0	5.0	1.0

a Free-space/single-source.

b Free-space/dual-source.

c Dielectric-inclusion/single-source.

d Multi-inclusion/single-source.

All training is performed using the Adam optimizer (with the learning rate of 5E-5 and decay coefficient of 0.9), implemented in Tensorflow and executed on Google Colab with Nvidia A100 GPU backend.

### Predicted results for free-space/single-source configuration

Here, we present the predicted results obtained using the proposed PI-DeepONet Framework. The prediction is performed over a temporal range of *t*∈[0,12] ns, and a spatial domain of *x*,*y*∈[-4,4]. To assess the baseline performance of the PI-DeepONet, wave propagation with a single source is first considered. A single Gaussian pulse used to initiate wave propagation is defined as

(Equation 9) $$U\left(x,y,0\right)=\exp\left(-\frac{{\left(x-x_{1}\right)}^{2}+{\left(y-y_{1}\right)}^{2}}{0.5}\right)\text{.}$$

**Figure 3:**
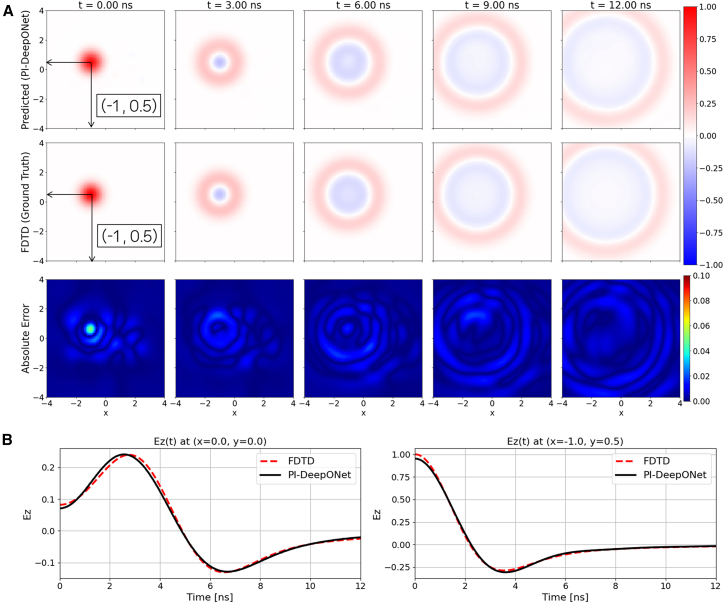
Free-space/single-source case with absorbing boundary condition (A) Spatial evolution of Ez predicted by PI-DeepONet (top row) compared with the FDTD reference (middle row) at selected time instants. The bottom row shows the absolute error map, |Ez_PI-DeepONet_ − Ez_FDTD_|. (B) Temporal Ez waveforms at two probe locations, (0, 0) and (−1, 0.5), comparing PI-DeepONet with FDTD.

For training, the center location of the Gaussian pulse (*x*_1_,*y*_1_) is randomly sampled from [–2, 2] in the x-direction and [–1, 1] in the y-direction. Therefore, the Branch Net takes the pulse-center coordinates [*x*_1_,*y*_1_] as its input, while the Trunk Net takes the spatiotemporal coordinates [*x*,*y*,*t*] as its input. Consequently, the trained model is expected to generalize to any source location within this predefined region during inference. However, note that this study does not support an arbitrary source type. Moreover, the free-space/single Gaussian pulse configuration is selected as the baseline case to verify the fundamental wave propagation characteristics of the proposed PI-DeepONet. Since this configuration represents a standard scenario without material discontinuities, only the ABC is briefly applied to emulate open-space propagation and suppress spurious reflections at the domain edges. In this configuration, a network with 20 hidden layers and 64 neurons per hidden layer is utilized (as summarized in Table 1). The model is trained for 100,000 epochs, and the total training time was approximately 366 min. Figure 3A illustrates the predicted field distributions obtained from PI-DeepONet alongside the corresponding FDTD reference and absolute error maps at representative time instants (t = 0, 3, 6, 9, and 12 ns). The predicted field patterns closely follow the FDTD results, accurately reproducing the outwardly propagating wavefronts with maximum absolute error remaining below 5.1%. Figure 3B presents the temporal profiles of *E*_*z*_(*t*) at two probe locations, (0, 0) and (−1, 0.5). The predicted time-domain responses show strong agreement with the FDTD reference across the entire simulation window, validating the model’s capability to accurately track field oscillations and amplitude variations at arbitrary points within the domain. These results collectively demonstrate that the PI-DeepONet can reliably reproduce transient wave propagation in free space, forming a foundation for subsequent evaluations under more complex scenarios. Note that to keep the article concise while still demonstrating the intended capability, we selected representative unseen configurations for each boundary condition setting. The additional prediction generated with the same model (different source/inclusion positions) are provided in the supplemental information (Figures S1–S7).

### Predicted results for free-space/dual-source configuration

Having validated the model in the single-source scenario, the model is further enhanced to test under a configuration where two spatially separated Gaussian sources are simultaneously introduced in the free-space domain. The corresponding initial electric field is given by:

(Equation 10) $$U\left(x,y,0\right)=\exp\left(-\frac{{\left(x-x_{1}\right)}^{2}+{\left(y-y_{1}\right)}^{2}}{0.25}\right)+\exp\left(-\frac{{\left(x-x_{2}\right)}^{2}+{\left(y-y_{2}\right)}^{2}}{0.25}\right)\text{.}$$

In this configuration, the wavefield exhibits more complex interference and superposition effects, making it a more challenging task for generalization. To accommodate the dual-source setup, the input space for the Branch Net is expanded to simultaneously encode both source locations (*x*_1_,*y*_1_) and (*x*_2_,*y*_2_). This extension enables the model to learn the joint influence of multiple excitations and their spatiotemporal interactions, which are commonly encountered in real-world EM applications. During the training, the first source location (*x*_1_,*y*_1_) is randomly sampled from the range [0, 2]×[0, 1], while the second source (*x*_2_,*y*_2_) is sampled from [–2, 0]×[–1, 0], ensuring spatial separation between the two emitters. The Branch Net takes the two pulse-center coordinates [*x*_1_,*y*_1_,*x*_2_,*y*_2_] as its input, while the Trunk Net takes the spatiotemporal coordinates [*x*,*y*,*t*] as its input. For this configuration, we employed a network consisting of 30 hidden layers with 64 neurons per layer (Table 1). The training is conducted for 100,000 epochs, requiring approximately 375 min in total. The inference is performed under the same grid used in the previous case.

**Figure 4:**
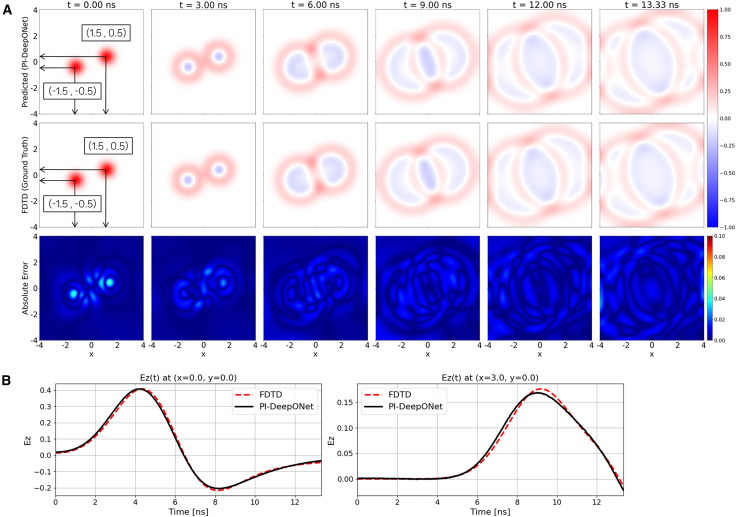
Free-space/dual-source case with reflecting boundary condition (A) Spatial evolution of Ez predicted by PI-DeepONet (top row) compared with the FDTD reference (middle row) at selected time instants. The bottom row shows the absolute error map, |Ez_PI-DeepONet_ − Ez_FDTD_|. (B) Temporal Ez waveforms at two probe locations, (0, 0) and (3, 0), comparing PI-DeepONet with FDTD.

Figure 4A presents the spatiotemporal wavefield evolution under reflecting (Neumann) boundary conditions. In this experiment, the simulation time is extended to 13.33 ns to clearly visualize the interaction between the outgoing waves and the domain boundaries. As the wavefronts generated by the two Gaussian sources expand outward, they eventually reach the boundaries and reflect back into the domain, resulting in characteristic standing-wave patterns and multi-layered interference structures. The corresponding absolute-error plots confirm that the deviation remains low with a maximum absolute error remaining below 5.2%, demonstrating that the network accurately models both the primary outward propagation and the subsequent boundary reflections. Figure 4B shows the temporal evolution at two representative probe locations inside the domain. The first probe is positioned near the center of the interference pattern, while the second probe is located close to the right boundary where reflected waves re-enter the domain. In both cases, the PI-DeepONet predictions exhibit strong agreement with the FDTD reference, accurately reproducing the amplitude modulation induced by the superposition of incident and reflected wavefronts.

**Figure 5:**
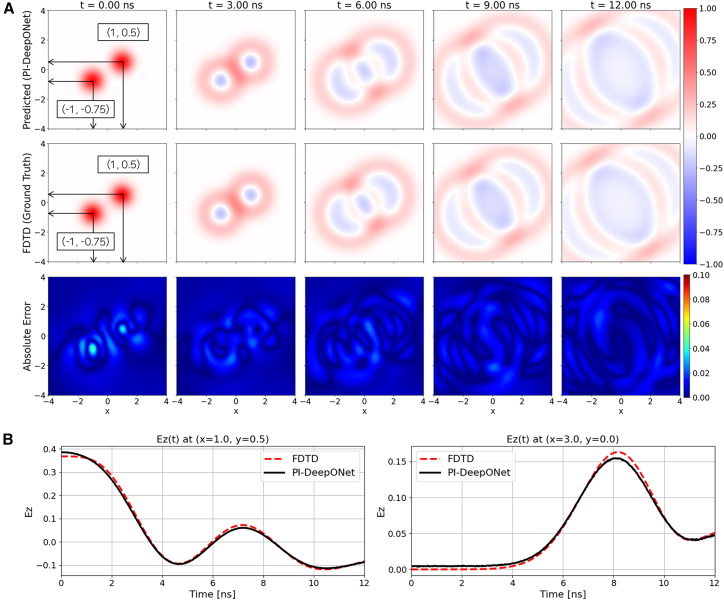
Free-space/dual-source case with absorbing boundary condition (A) Spatial evolution of Ez predicted by PI-DeepONet (top row) compared with the FDTD reference (middle row) at selected time instants. The bottom row shows the absolute error map, |Ez_PI-DeepONet_ − Ez_FDTD_|. (B) Temporal Ez waveforms at two probe locations, (1, 0.5) and (3, 0), comparing PI-DeepONet with FDTD.

To further evaluate the generalization capability of the proposed PI-DeepONet, the dual-source configuration is additionally tested under ABC. The initial condition is the same as (10), but the denominators inside the exponential terms are changed from 0.25 to 0.5. Importantly, the source locations used during inference differ from those in reflecting boundary condition, thereby enabling a direct assessment of the model’s ability to generalize to unseen source configurations within the continuous input space of the Branch Net. The same network configuration as the reflecting boundary condition is utilized with a total training time of 382 min. Figure 5A presents the predicted electric field snapshots generated by PI-DeepONet, the corresponding FDTD ground truth, and the absolute error distributions at several time instants. Despite the change in source locations, the PI-DeepONet accurately reconstructs the propagating wavefronts and the continuously evolving interference patterns formed by the two emitters with a maximum absolute error rate under 4.8%. Figure 5B shows the temporal waveforms of *E*_*z*_(*t*) recorded at two probe points placed at center of an emitter (1, 0.5), and at a location further from the emitters. Across both probes, the PI-DeepONet prediction closely matches the FDTD reference, accurately capturing the amplitude behavior induced by the superposition of the two Gaussian pulses.

### Predicted results for dielectric-inclusion/single-source configuration

Accurate field prediction in inhomogeneous media is essential for modeling practical EM environments, where spatial variations in material properties are common. These variations often lead to complex wave phenomena, such as scattering and refraction, which are not present in ideal free-space settings. To verify the robustness of the PI-DeepONet under such conditions, a scenario involving a single-Gaussian source configuration in inhomogeneous media is considered. Unlike the previous free-space configurations, where only the source location varied, this setting introduces an additional degree of freedom by allowing both the source position and the dielectric block position to vary across inference samples. This increased variability enables a more comprehensive assessment of the model’s generalization performance under spatial heterogeneity. During training, the center of the Gaussian source is randomly sampled from the region −2≤*x*_1_ ≤ 2 and −1≤*y*_1_ ≤ 0, while the center of the dielectric block is independently varied within the domain from the region −2≤*x*_3_ ≤ 2 and 2 ≤ *y*_3_ ≤ 3. Therefore, the Branch Net takes the single pulse-center coordinates [*x*_1_,*y*_1_] as its input, while the Trunk Net takes both the spatiotemporal coordinates and dielectric block-center coordinates [*x*,*y*,*t*,*x*_3_,*y*_3_] as its input. Since the dielectric block has a fixed size of 4 m (width) by 2m (height) and a relative permittivity (*ε*_*r*_) of 4, the material property and size parameters are not included in the present work. The initial field distribution is defined by the same Gaussian function as (9). All configurations from now on use the same network with an identical number of hidden layers and neurons per layer, as summarized in Table 1.

**Figure 6:**
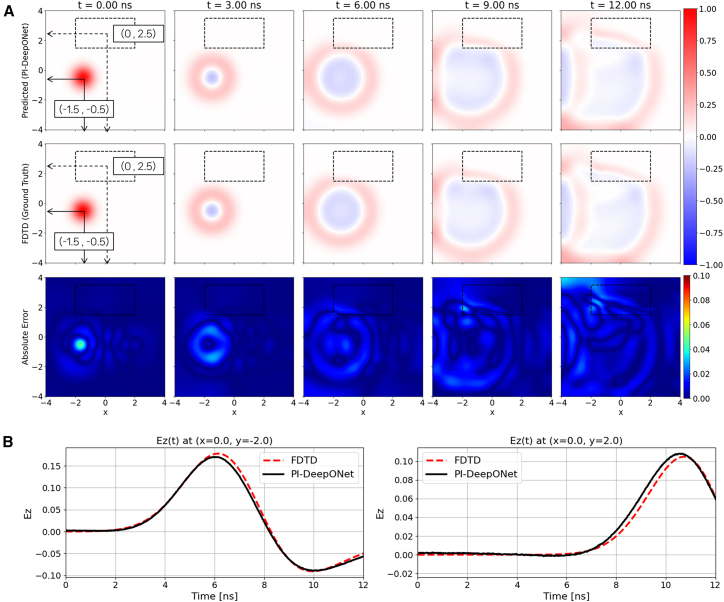
Dielectric-inclusion/single-source case with reflecting boundary condition (A) Spatial evolution of Ez predicted by PI-DeepONet (top row) compared with the FDTD reference (middle row) at selected time instants. The bottom row shows the absolute error map, |Ez_PI-DeepONet_ − Ez_FDTD_|. (B) Temporal Ez waveforms at two probe locations, (0, −2) and (0, 2), comparing PI-DeepONet with FDTD.

In this scenario, the dielectric block is positioned at (*x*_3_,*y*_3_) = (0, 2.5), as indicated by the dashed rectangle (Figure 6A). The Gaussian source is placed at (−1.5, −0.5), generating a wavefront that interacts with the inclusion. As the wave impinges on the dielectric block, noticeable refraction, slowing of the wavefront, and partial reflection occur, forming field distortions that are absent in free-space propagation. The PI-DeepONet accurately reproduces these behaviors across all time instants shown, successfully capturing the curvature changes and amplitude variations resulting from the dielectric contrast. It is observed that the regions near the dielectric block–where wave refraction and partial reflections occur–exhibit slightly larger prediction errors compared to the free-space background. This behavior is expected due to the increased field complexity at material interfaces, which demands higher model expressiveness. While further increasing the network capacity (e.g., depth or width of the Branch and Trunk Nets) may improve the accuracy in these regions, such extensions are constrained by available computational resources in the current implementation. Figure 6B shows the temporal evolution of the electric field measured at two probe points: one located outside the dielectric block (0, −2), and the other placed inside the dielectric block (0, 2), where the refracted and partially reflected wave converge. At the lower probe position, where the field is dominated by direct propagation from the source, the predicted amplitude agrees nearly perfectly with the ground truth. At the upper probe, the waveform exhibits distortions due to the dielectric-induced refraction and delayed arrival of the wavefront; nevertheless, the PI-DeepONet successfully tracks these features and retains high accuracy even in regions influenced by complex wave-medium interactions.

**Figure 7:**
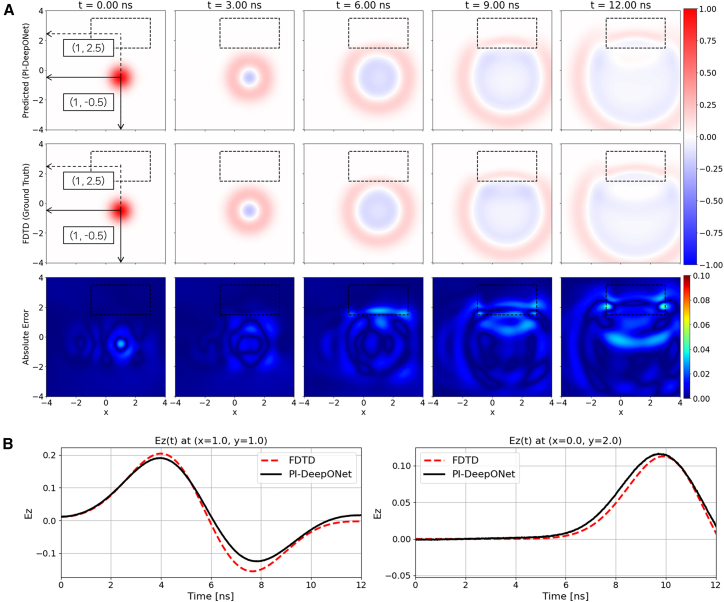
Dielectric-inclusion/single-source case with absorbing boundary condition (A) Spatial evolution of Ez predicted by PI-DeepONet (top row) compared with the FDTD reference (middle row) at selected time instants. The bottom row shows the absolute error map, |Ez_PI-DeepONet_ − Ez_FDTD_|. (B) Temporal Ez waveforms at two probe locations, (1, 1) and (0, 2), comparing PI-DeepONet with FDTD.

Figure 7A illustrates the predicted electric field maps, the corresponding FDTD reference, and the absolute error distributions for the scenario in which both the source and the dielectric inclusion are placed at locations different from those used in the reflecting boundary condition. In this example, the source is positioned at (1, −0.5) and the dielectric block is located at (1, 2.5). Under ABC, the waves are effectively attenuated at the domain edges, preventing the formation of boundary reflections. The absolute error remains low throughout the domain (maximum absolute error of 5.8%, which is similar to that of the reflecting boundary condition), confirming that the operator-learning model generalizes effectively. Figure 7B presents the time-domain waveforms recorded at two probe locations: a region located between the source and the dielectric block (1, 1), where the strongest wave-medium interactions occur, and a point located inside the dielectric inclusion (0, 2). At both probe points, the PI-DeepONet predictions closely follow the FDTD reference. The waveform at (1, 1) captures the amplitude variations generated by multiple interaction mechanisms, whereas the waveform at (0, 2) accurately reflects the slower wave propagation similar to the previous case.

Although fixed rectangular inclusions provide a clean and controlled benchmark, introducing irregularly shaped media can better reflect complex real-world environments. To assess the robustness of our approach beyond axis-aligned rectangles, we conducted an additional test using an amoeba-shaped (irregular) dielectric inclusion under the absorbing boundary condition, as shown in Figure S8. In this test, we used the same trained network and evaluated two cases in which the irregular inclusion was placed at different positions. As evidenced by the predicted field snapshots and the corresponding absolute-error maps in Figure S8, the error patterns and magnitudes remain comparable to those observed in the rectangular-inclusion cases, indicating that the proposed PI-DeepONet generalizes well to irregular interfaces without a noticeable degradation in accuracy.

### Predicted results for multi-inclusion/single-source configuration

Building upon the single-inclusion case, we next consider a more challenging scenario in which the propagating wave interacts with two dielectric inclusions. The presence of multiple scatterers significantly increases the complexity of the wavefield, as the incident wave undergoes sequential refractions and partial reflections at different interfaces. These interactions give rise to multi-path propagation and compounded interference patterns that are substantially more intricate than those observed in the single-inclusion configuration. Evaluating the PI-DeepONet under this extended setting allows us to assess its capability to generalize across environments featuring multiple material discontinuities, which more closely resemble real-world EM propagation scenarios. In this subsection, a single Gaussian source is also used while the positions of the two dielectric inclusions are varied independently during training, enabling a comprehensive examination of the model’s robustness and predictive accuracy under multi-scatterer conditions.

**Figure 8:**
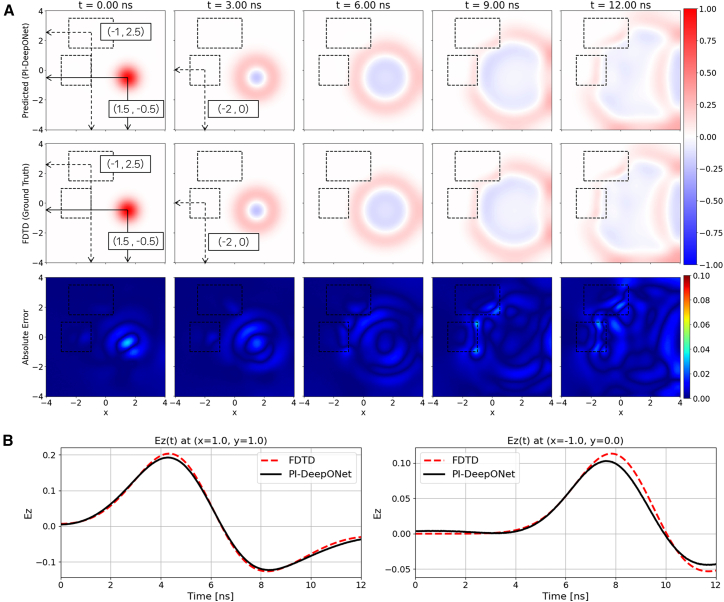
Multi-inclusion/single-source case with reflecting boundary condition (A) Spatial evolution of Ez predicted by PI-DeepONet (top row) compared with the FDTD reference (middle row) at selected time instants. The bottom row shows the absolute error map, |Ez_PI-DeepONet_ − Ez_FDTD_|. (B) Temporal Ez waveforms at two probe locations, (1, 1) and (−1, 0), comparing PI-DeepONet with FDTD.

**Figure 9:**
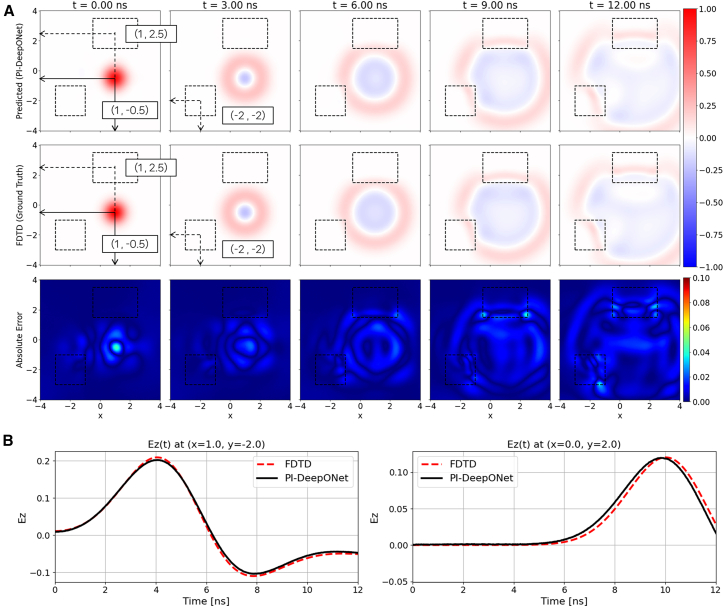
Multi-inclusion/single-source case with absorbing boundary condition (A) Spatial evolution of Ez predicted by PI-DeepONet (top row) compared with the FDTD reference (middle row) at selected time instants. The bottom row shows the absolute error map, |Ez_PI-DeepONet_ − Ez_FDTD_|. (B) Temporal Ez waveforms at two probe locations, (1, −2) and (0, 2), comparing PI-DeepONet with FDTD.

As in the previous configurations, both Neumann boundary conditions and absorbing boundary conditions are employed to evaluate the generalization capability of the proposed PI-DeepONet under different wave–boundary interactions. The medium now contains two dielectric inclusions with different sizes: Block 1 has dimensions of 3 m × 2 m, while Block 2 measures 2 m × 2 m. During training, the center of Block 1 is constrained to the line *y*_3_ = 2.5 with its horizontal position uniformly sampled from −2≤*x*_3_ ≤ 2. For Block 2, the center is fixed at *x*_4_ = −2, while its vertical position is sampled from −2≤*y*_4_ ≤ 0 (*x*_4_ and *y*_4_ are not shown in Trunk Net in Figure 2). The location of the Gaussian pulse is independently varied within the range −2≤*x*_1_ ≤ 2 and −1≤*y*_1_ ≤ 0. The Branch Network, therefore, takes the single pulse-center coordinates [*x*_1_,*y*_1_] as its input, while the Trunk Net takes the spatiotemporal coordinates and two dielectric block-center [*x*,*y*,*t*,*x*_3_,*y*_3_,*x*_4_,*y*_4_] as its input. The resulting prediction performance for unseen combinations of source and inclusion positions, under both Neumann and ABC, is illustrated in Figures 8 and 9.

Figure 8A presents the predicted electric field snapshots from PI-DeepONet, the corresponding FDTD references, and the absolute error maps for the case with Neumann boundary conditions. In this test scenario, the Gaussian source is positioned at (1.5, −0.5), while the two dielectric inclusions are located at (−1, 2.5) for Block 1 and (−2, 0) for Block 2. Due to the reflecting nature of the Neumann boundaries, the outward-propagating wavefronts undergo multiple reflections at the domain edges, leading to complex standing-wave patterns and multi-path interference as the waves interact not only with the inclusions but also with the reflecting walls. Block 1, located in the upper region of the domain, induces clear refraction and wave delay, while Block 2 introduces secondary distortions as the wavefront grazes or partially enters its interior. The PI-DeepONet predictions remain in close agreement with the FDTD ground truth across all time steps, successfully reconstructing the compounded interactions between the two inclusions and the reflecting boundaries. As expected, the absolute error becomes slightly elevated near the boundaries of the dielectric blocks–where refraction, partial reflections, and abrupt spatial gradients occur–but remains low elsewhere in the domain. Figure 8B shows the temporal waveforms measured at two probe locations. Similar to the previous configuration, the PI-DeepONet predictions closely follow the FDTD reference at both probe points.

Figure 9A illustrates the field evolution when the same dual-inclusion configuration is evaluated under ABC. With boundary reflections eliminated, the propagating wavefronts interact only with the two dielectric inclusions, enabling a clearer observation of refraction and localized amplitude distortion around each block. The absolute error maps show that deviations remain small across the domain, with slightly higher errors concentrated near the dielectric interfaces where rapid spatial transitions occur. Figure 9B presents the temporal response at two probe locations: (1, −2), situated near the lower region where the wavefront arrives early with minimal scattering, and (0, 2) located near the upper part of the domain where the refracted wave interacts with Block 1. In both cases, the PI-DeepONet closely follows the FDTD reference, capturing the trend and amplitude variations with high fidelity.

These results demonstrate that the proposed PI-DeepONet retains strong predictive accuracy not only in a simple only-source configuration, but also even in multi-scatterer environments, capturing both spatial and temporal field behaviors under diverse source and boundary configurations. This validates the robustness and generalization capability of the model across increasingly complex electromagnetic scenarios.

## Discussion

Computational efficiency is another important consideration when comparing the trained PI-DeepONet with conventional solvers. Once trained, the PI-DeepONet allows for the rapid prediction of a single temporal snapshot at any arbitrary time instance without requiring the full simulation history. In contrast, FDTD must compute the entire wave evolution sequentially from *t* = 0 up to the desired time, which requires substantially more computation. To evaluate the computational efficiency of the proposed PI-DeepONet model, a comparison is made against an FDTD solver under various spatial grid resolutions, as summarized in Table 3. For PI-DeepONet, we report both the single-instance inference time (direct prediction of one snapshot at an arbitrary queried time) and the total predicted time (evaluating all 601-time steps). For FDTD, we report the total computation time required to time-march over the full window. Two spatial grid settings are considered: a coarse grid with *N*_*x*_ = *N*_*y*_ = 81, and a finer grid with *N*_*x*_ = *N*_*y*_ = 241, while the temporal resolution (*N*_*t*_) remains fixed to 601 points. For the coarser grid of 81 × 81, the total prediction time of PI-DeepONet ranges from 2.43 to 3.36 s, depending on the complexity of the scenario, which is up to 5 times faster than the FDTD solver (12.43–16.27 s). As the grid becomes denser (301 × 301), the FDTD runtime increases significantly, rising to 112.71 to 123.34 s, due to the inherent sequential update nature of the time-domain solver. In contrast, the total inference time of PI-DeepONet increases modestly, remaining within 7.43 s (up to 15 times faster than FDTD), highlighting the scalability advantage of the model in high-resolution settings. Notably, the single-instance (random-access) inference time remains extremely small – below 0.015 s for both the coarser and finer grids – demonstrating that PI-DeepONet can directly query arbitrary time snapshots with negligible latency. Importantly, the root-mean-squared error (RMSE) values remain consistently low across all cases and grid settings, demonstrating the robustness and generalization capacity of PI-DeepONet. Even with a 3-fold increase in grid density, the RMSE variation is marginal (shows even lower RMSE in a complicated case, e.g., from 6.17E-3 to 4.09E-3 in the multi-inclusion/single source case), indicating that prediction accuracy is preserved regardless of the grid resolution. Additional quantitative error breakdowns, including the interface-local RMSE (near the inclusion boundary) versus the total RMSE over the full domain, are provided in Table S1. In addition, Figure S9 summarizes the error histograms of the total time-averaged RMSE and MAE over randomly sampled source and inclusion locations in dielectric-inclusion/single source configuration for the representative case to further demonstrate the model’s consistency across configurations.

**Table 3:** Comparison of total prediction time of PI-DeepONet, FDTD computation time, and RMSE across different scenarios and spatial resolutions

Configuration		N_x_ = N_y_ = 81, N_t_ = 601				N_x_ = N_y_ = 241, N_t_ = 601			
		PI-DeepONet		FDTD	RMSE	PI-DeepONet		FDTD	RMSE
		Single instance	Total			Single instance	Total		
Case 1	ABC	0.0047s	2.43s	12.43s	3.93E-3	0.0085s	5.37s	113.23s	3.78E-3
Case 2	ABC	0.0051s	3.22s	16.27s	4.87E-3	0.012s	7.11	112.71s	4.22E-3
	RBC	0.0048s	3.12s	12.55s	6.78E-3	0.011s	7.09	114.52s	6.61E-3
Case 3	ABC	0.0052s	3.21s	13.61s	7.28E-3	0.012s	7.28	123.34s	6.41E-3
	RBC	0.0054s	3.36s	14.14s	6.8E-3	0.012s	7.43	122.62s	7.24E-3
Case 4	ABC	0.0055s	3.35s	12.72s	6.17E-3	0.013s	7.34	116.79s	4.09E-3
	RBC	0.0054s	3.34s	12.8s	6.7E-3	0.012s	7.24	116.69s	5.82E-3

Beyond grid-resolution robustness, an equally important question for time-domain EM surrogates is whether they maintain physical consistency and stability over total temporal evolution. For the scalar time-domain Helmholtz considered in this work, a natural stability indicator is an energy functional of the form

(Equation 11) $$E\left(t\right)=\frac{1}{2}\int \int_{\Omega }\left({\left(\frac{\partial \hat{U}}{\partial t}\right)}^{2}+c^{2}\left[{\left(\frac{\partial \hat{U}}{\partial x}\right)}^{2}+{\left(\frac{\partial \hat{U}}{\partial y}\right)}^{2}\right]\right)\;dxdy\text{.}$$

**Figure 10:**
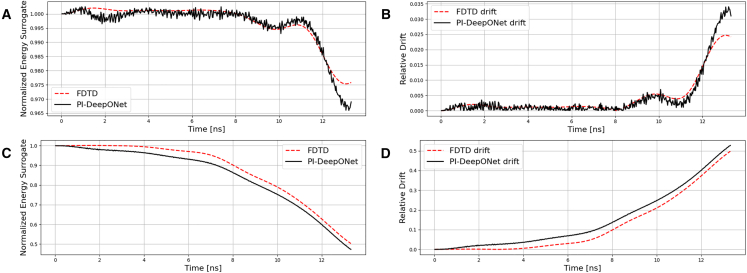
Verification of the numerical stability of the simulations (A) Normalized energy surrogate and (B) relative energy drift calculated in free-space/dual-source case under reflecting boundary condition. (C) Normalized energy surrogate and (D) relative energy drift calculated in a multi-inclusion/single-source case under absorbing boundary condition.

In stable time-marching solutions, such an indicator is expected to remain bounded and to exhibit only limited drift, whereas numerical instabilities typically manifest as an unphysical growth of this global measure. To quantify full-time stability in a normalized manner, we also report the relative energy drift

(Equation 12) $$D\left(t\right)=\frac{\left|E\left(t\right)-E\left(t_{0}\right)\right|}{E\left(t_{0}\right)}\text{,}$$

where *t*_0_ is the first evaluated time index (i.e., *t*_0_ = 0). In the present wave-propagation setting, *E*(*t*) (and therefore *D*(*t*)) depends on the imposed boundary condition. Under reflecting (Neumann) boundaries, the wave energy is ideally conserved within the computational domain, so *E*(*t*) should remain approximately constant and *D*(*t*) should stay small; in practice, a limited drift is still expected due to spatial–temporal discretization and the discrete implementation of boundary conditions. Consistent with this, in the free-space dual-source case (which is chosen to be a representative case for the free-space case) with reflecting boundaries (as shown in Figures 10A and 10B), both FDTD and PI-DeepONet energy-surrogate curves nearly overlap over the full time window, and both remain bounded close to its initial value. The corresponding *D*(*t*) for PI-DeepONet also closely tracks that of FDTD and remains at only a few-percent level, indicating stable long-time evolution without spurious energy growth. In contrast, under ABC, energy is intentionally allowed to leave the domain; accordingly, *E*(*t*) is expected to decrease over time even for a physically stable simulation, and *D*(*t*) can grow because it measures deviation from the initial energy, not numerical instability. Therefore, for ABC, we assess stability by verifying that (i) *E*(*t*) decays in a physically consistent and bounded manner and (ii) the decay trend calculated by PI-DeepONet closely follows the FDTD reference. As shown in the representative multi-inclusion single-source case with ABC (shown in Figures 10C and 10D), the PI-DeepONet and FDTD energy-surrogate curves again exhibit very similar decay profiles, with only minor deviations while remaining bounded throughout the entire temporal evolution; the associated drift curves show a comparable growth trend because both methods reflect the intended energy outflow through the absorbing boundary.

To quantify whether the prediction error is concentrated near material discontinuities, we compare the PI-DeepONet prediction $\hat{U}\left(x_{i},y_{j},t_{n}\right)$ with the FDTD reference *U*(*x*_*i*_,*y*_*j*_,*t*_*n*_) on the same uniform Cartesian grid. The pointwise error at a discrete time *t*_*n*_ and grid point (*x*_*i*_,*y*_*j*_) is defined as

(Equation 13) $$e\left(t_{n},x_{i},y_{j}\right)=\hat{U}\left(x_{i},y_{j},t_{n}\right)-U\left(x_{i},y_{j},t_{n}\right)\text{.}$$

For each dielectric inclusion, we define an interface band Ω_*int*_ as a region surrounding the material boundary Γ. In practice, we construct Ω_*int*_ using a signed-distance function *d*(*x*,*y*) to the inclusion boundary (negative inside, positive outside, and zero on Γ):

(Equation 14) $$\Omega_{\mathrm{int}}=\left\{\left(x_{i},y_{j}\right)\in \Omega :\left|d\left(x_{i},y_{j}\right)\leq \delta \right|\right\},\delta =N_{band}\;\max\left(\Delta x,\Delta y\right)\text{,}$$

where Δ*x* and Δ*y* are the spatial grid spacing, and *N*_*band*_ is a prescribed number of grid cells (fixed across cases). For multi-inclusion configurations, Ω_*int*_ is taken as the union of the interface bands of all inclusions. The “total” region corresponds to the full computational domain Ω.

At each time snapshot *t*_*n*_, we compute the RMSE over the region by averaging over the grid points that belong to Ω_*s*_:

(Equation 15) $$RMSE_{\Omega_{s}}\left(t_{n}\right)=\sqrt{\frac{1}{N_{s}}\sum_{\left(i,j\right)\in \Omega_{s}}e{\left(x_{i},y_{j},t_{n}\right)}^{2}}\text{,}$$

where *N*_*s*_ is the number of grid points in Ω_*s*_. We then report both the time-averaged and time-maximum RMSE over the full temporal window as

(Equation 16) $${\bar{RMSE}}_{\Omega_{s}}=\frac{1}{N_{s}}\sum_{n=1}^{N_{t}}RMSE_{\Omega_{s}}\left(t_{n}\right)\text{,}$$

(Equation 17) $$RMSE_{\Omega_{s}}^{\max}=\max_{n}RMSE_{\Omega_{s}}\left(t_{n}\right)\text{.}$$

Table S1 compares interface-local versus total RMSE under different boundary conditions and inclusion configurations. For the single-inclusion cases, the interface-local RMSE is consistently larger than the total RMSE, indicating that the dominant errors are concentrated near the dielectric interface where the field exhibits sharper spatial variations. For the multi-inclusion configurations, this trend persists under reflecting boundary conditions. Under absorbing boundary conditions, however, the total RMSE can exceed the interface-local RMSE, suggesting that the error is not solely confined to immediate interface neighborhoods but can be distributed throughout the bulk due to more complex multi-scattering interference patterns. Overall, these results quantitatively support that interface regions are often the most challenging parts of the domain, while also clarifying when bulk contributions become significant.

To further assess the robustness of the proposed model across different geometric configurations, we provide histogram summaries of the prediction error over randomly sampled test cases. We performed a representative subset analysis by randomly sampling configurations within the same location ranges used during training. As a representative benchmark, the single dielectric inclusion with single-source scenario under Neumann (reflecting) boundary conditions is considered. For each sampled case, the center location of the rectangular dielectric inclusion is randomly drawn within the range [−2, 2] × [2, 3], and the source (pulse) center location is randomly drawn within [−2, 2] × [−1, 0]. We generate 50 such configurations. For each configuration, PI-DeepONet prediction and the corresponding FDTD reference are computed on the same space-time grid and then evaluated total time-averaged error metrics by aggregating the discrepancy over the full spatial domain and over all time steps. Figure S9 shows histograms of the total time-averaged RMSE and MAE over the 50 randomly sampled configurations. The resulting distributions are unimodal and concentrated within a relatively narrow range, indicating that the model maintains a consistent accuracy level across diverse source and inclusion placements rather than relying on a small subset of favorable cases. A mild right tail is observed, reflecting a limited number of more challenging configurations in which the interaction between the scattered field and reflecting boundaries produces more complex interference patterns. Overall, these histogram summaries provide an additional dataset-level validation that the proposed PI-DeepONet exhibits stable performance across the sampled parameter space.

These aforementioned analyses validate that PI-DeepONet not only offers significant speedup over FDTD, particularly at larger spatial scales, but also maintains comparable accuracy, making it a viable alternative for real-time or large-scale EM field modeling.

In modern time-domain computational electromagnetics, long-term robustness is often tied to maintaining key physical constraints and material fidelity beyond matching the primary wave equation. For example, advanced FDTD developments explicitly preserve discrete Gauss’s laws (i.e., divergence constraints) to suppress nonphysical charge/flux artifacts that can accumulate in semi-implicit updates over long simulations, as demonstrated by divergence-preserving HIE-FDTD formulations with open-region truncation via CFS-PML.^34^ In parallel, accurate modeling of complex dispersive media has been enabled by unified multi-term constitutive frameworks (e.g., multiterm modified Lorentz models) combined with auxiliary differential equation (ADE) updates within unconditionally stable FDTD variants, allowing Debye/Drude/Lorentz-type responses to be incorporated without prohibitive time-step restrictions.^35^

In the present work, we deliberately focus on a reduced, TM_z_ wave propagation setting and enforce physics through a scalar, second-order time-domain wave equation for a single field component (interpreted as E_z_). Within this scalar TM_z_ formulation, divergence constraints of the full vector Maxwell system are less directly exposed because the surrogate does not explicitly evolve all vector components (E, H field) and their coupled curl updates, where discrete violations of Gauss’s laws can accumulate over long time integration in semi-implicit schemes. Nevertheless, the broader perspective from advanced time-domain solvers remains highly relevant: if the surrogate is extended toward full-wave vector Maxwell formulations (e.g., predicting multiple field components and enforcing coupled first-order curl equations), then divergence-consistency regularization or constraint-preserving discretization becomes important to mitigate spurious divergence drift and the emergence of unphysical field modes. For open-region problems, we currently employ a first-order absorbing boundary condition (ABC) as a lightweight radiation model at the truncated computational boundary. As a next step, this treatment can be upgraded to perfectly matched layers (PML) – including convolutional forms such as CFS-PML – by augmenting the governing equations with PML auxiliary variables in a buffer region and incorporating the corresponding residual terms into the training loss. More broadly, aligning PI-DeepONet with state-of-the-art time-domain CEM/FDTD suggests a systematic roadmap: (i) moving from a scalar TM_z_ surrogate to a multi-output vector Maxwell surrogate with coupled first-order curl residuals, (ii) extending beyond nondispersive *ε*_*r*_(*x*,*y*) to dispersive/absorptive materials by introducing ADE-type internal state variables that are learned and constrained alongside the fields. These extensions would preserve the operator-learning advantages of PI-DeepONet while bringing its physical fidelity closer to modern divergence-preserving and dispersive-material FDTD methodologies.

In summary, this work establishes a physics-informed DeepONet as an efficient surrogate for time-domain 2D EM propagation that generalizes across both excitation and medium configurations. Across free-space single/dual source cases and dielectric-inclusion scenarios, the proposed model closely matches FDTD while maintaining low error and substantially reducing inference time. This generalization is enabled by the branch–trunk operator formulation (conditioning on source/material descriptors while querying spatiotemporal coordinates) together with physics-based training constraints, allowing accurate inference for unseen source positions and inclusion placements without retraining. Building on these results, several practical extensions remain. First, we will extend the framework to dispersive media, where the constitutive relations introduce an additional burden. Moreover, enabling inference under an arbitrary number of sources and an arbitrary number and shape of inclusions would also better reflect real-world wave environments. Second, we plan to pursue inverse-problem formulations, in which measured (or simulated) waveforms at the final time are used to infer the underlying scenario parameters, such as pulse location, dielectric-block location, and block permittivity. Finally, for inclusion problems, further work is needed to reduce localized errors near material interfaces; governing relations effectively become more challenging to satisfy due to abrupt parameter changes and sharper field gradients. Two practical strategies are particularly promising. First, we can place collocation points more densely near the inclusion boundary, i.e., concentrate training points within a thin band around the interface rather than sampling them uniformly over the entire domain. This increases the model’s exposure to the most difficult regions and encourages it to resolve boundary-localized features more accurately. Second, we can penalize boundary-region errors more strongly by assigning larger weights to the PDE and constitutive residuals evaluated near the interface (or by increasing these weights when discontinuity-induced residuals become dominant). In effect, the optimizer is explicitly driven to reduce interface-localized mismatch, which is often under-emphasized when losses are averaged over the full spatiotemporal domain. Together, these modifications are expected to improve accuracy in high-contrast inclusion settings while preserving the operator-learning efficiency and fast inference of PI-DeepONet.

### Limitations of the study

Although this study presents a PI-DeepONet that predicts spatiotemporal transient wave fields from compact scenario descriptors that can be applied to various environments, several limitations should be noted. First, we restrict attention to nondispersive media and a reduced TM_z_ scalar-wave formulation and therefore do not capture dispersive constitutive behavior that introduces temporal memory and increased stiffness. Second, the present results target the forward problem (field prediction given scenario parameters) rather than inverse formulations that infer unknown parameters from observed waveforms. Furthermore, in dielectric-inclusion cases, errors can be more localized near material interfaces, where abrupt coefficient changes and sharper field gradients make the governing relations harder to satisfy uniformly across the domain. These limitations motivate extensions that incorporate dispersive models, enable inverse-problem settings for estimating source and inclusion parameters, and reduce interface-localized errors using interface-aware training strategies such as band-focused sampling or region-weighted physics constraints.

## Resource availability

### Lead contact

Requests for further information and resources should be directed to and will be fulfilled by the lead contact, Sun K. Hong (shong215@ssu.ac.kr).

### Materials availability

This study did not generate new unique reagents.

### Data and code availability

• Data reported in this article will be shared by the lead contact upon request.
• Some of the original codes are available in this article’s supplemental information. Requests for additional codes are available from the lead contact upon request.
• Any additional information required to reanalyze the data reported in this article is available from the lead contact upon request.

## Acknowledgments

This study was supported in part by the Institute of Information & Communications Technology Planning & Evaluation (IITP) grant funded by the Korea government (10.13039/501100014188MSIT) (RS-2024-00393808, Efficient Design of RF Components and Systems Based on Artificial Intelligence), and in part by the 10.13039/501100001321National Research Foundation (NRF) of Korea [Grant 501100003725].

## Author contributions

Conceptualization, S.O. and S.K.H.; methodology, S.O.; investigation, S.O.; writing – original draft, S.O.; writing – review and editing, S.O., E.L., and S.K.H.; funding acquisition, S.K.H.; resources, S.O., and S.K.H.; supervision, E.L. and S.K.H.

## Declaration of interests

The authors declare no competing interests.

## STAR★Methods

### Key resources table

**Table:** 

REAGENT or RESOURCE	SOURCE	IDENTIFIER
**Software and algorithms**		

Google Colab	Google	https://colab.research.google.com/

### Experimental model and study participant details

This study employs Google Colab to implement and simulate PI-DeepONet as well as reference FDTD.

### Method details

The precise details of all the procedures are provided in the manuscript and the original code for PI-DeepONet is provided in supplemental information.

### Quantification and statistical analysis

Figures shown in the main text were produced by Google Colab.
