# Supplementary material for: Physic-informed deep operator networks for modeling 2D time-domain electromagnetic wave propagation in various media
Source: iScience. 2026 Feb 18;29(3):115076. doi: 10.1016/j.isci.2026.115076 (PMC12964304; doi:10.1016/j.isci.2026.115076)
Supplement: Document S1. Figures S1–S9, Table S1, and Methods S1–S4 [file mmc1.pdf]

iScience, Volume *29*

Supplemental information

Physic-informed deep operator networks for modeling 2D time-domain electromagnetic wave propagation in various media

Sooyoung Oh, EungKyu Lee, and Sun K. Hong

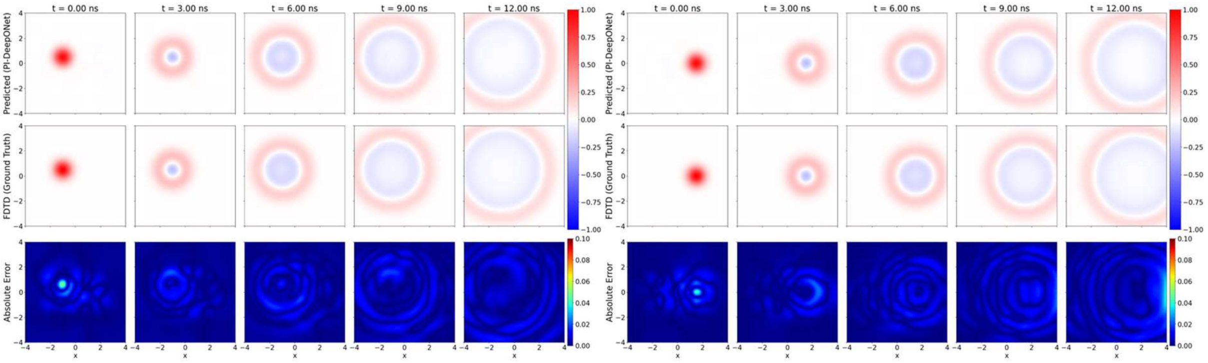

Figure. S1. Predicted field for free-space/single-source configuration (Reflecting BC) in two different source locations.

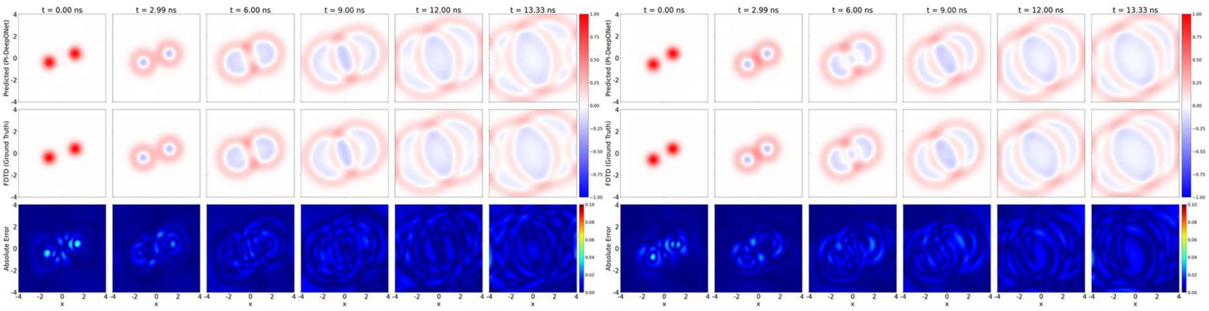

Figure. S2. Predicted field for free-space/dual-source configuration (Reflecting BC) in two different source locations.

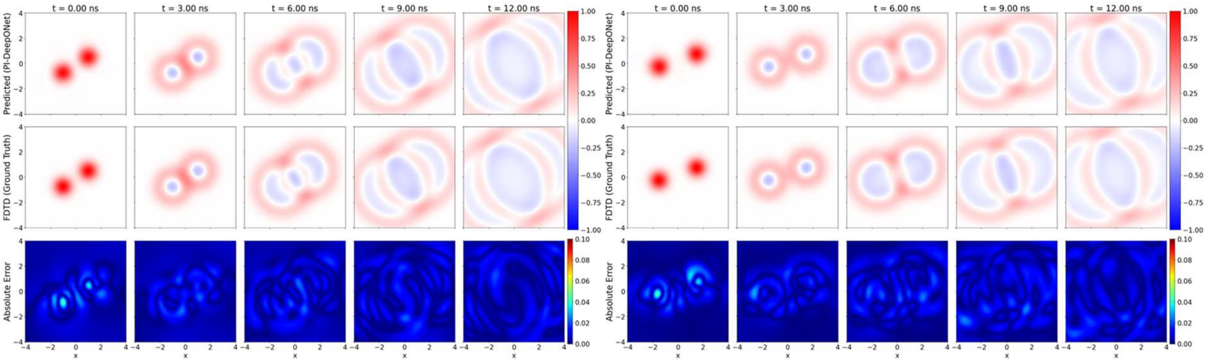

Figure. S3. Predicted field for free-space/dual-source configuration (ABC) in two different source locations.

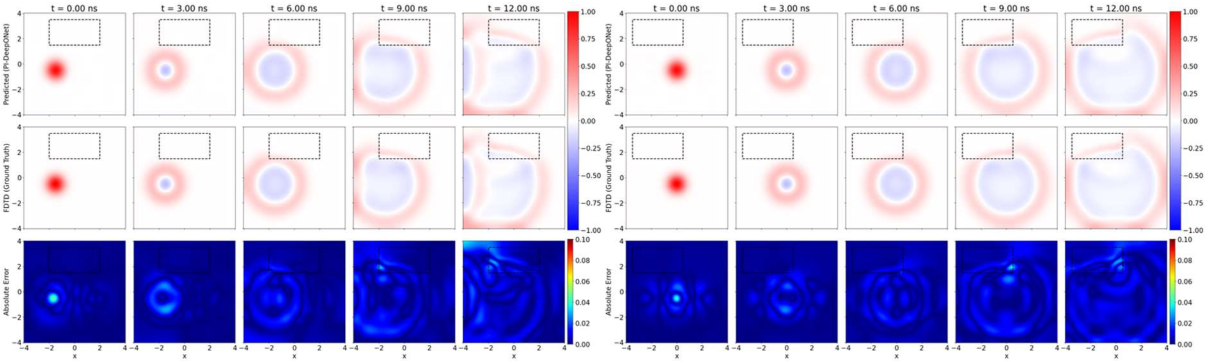

Figure. S4. Predicted field for dielectric-inclusion/single-source configuration (Reflecting BC) in two different source and inclusion locations.

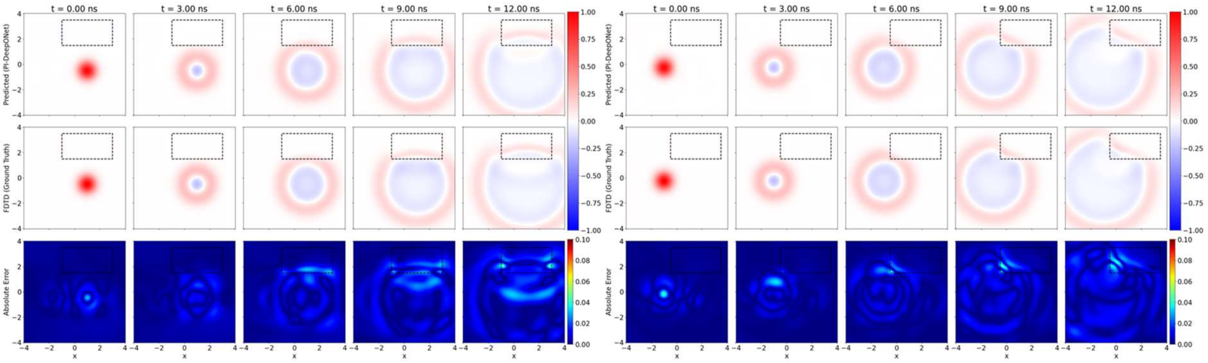

Figure. S5. Predicted field for dielectric-inclusion/single-source configuration (ABC) in two different source and inclusion locations.

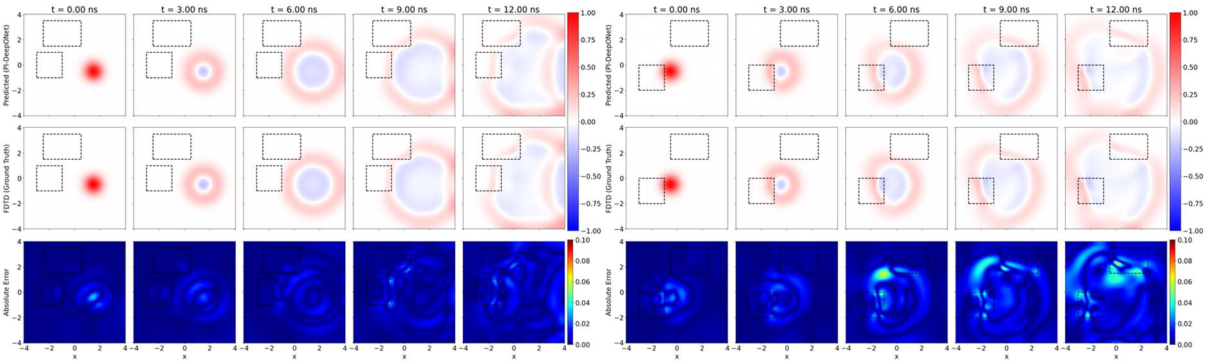

Figure. S6. Predicted field for multi-inclusion/single-source configuration (Reflecting BC) in two different source and inclusion locations.

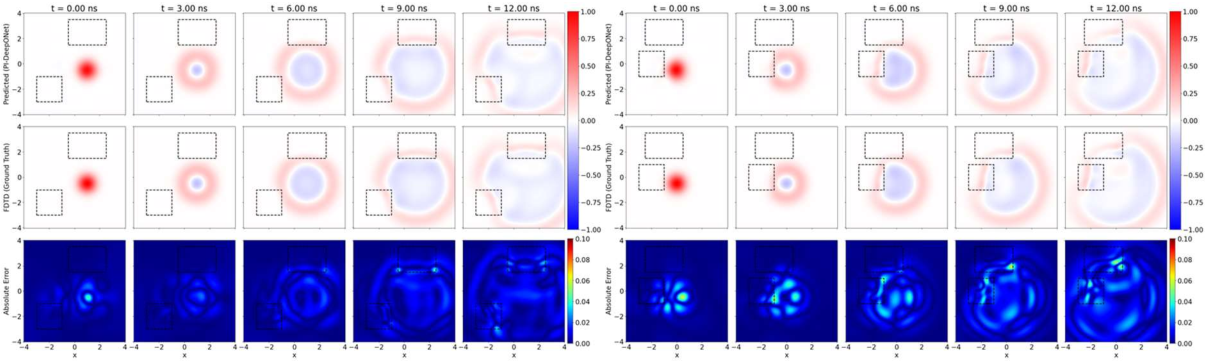

Figure. S7. Predicted field for multi-inclusion/single-source configuration (ABC) in two different source and inclusion locations.

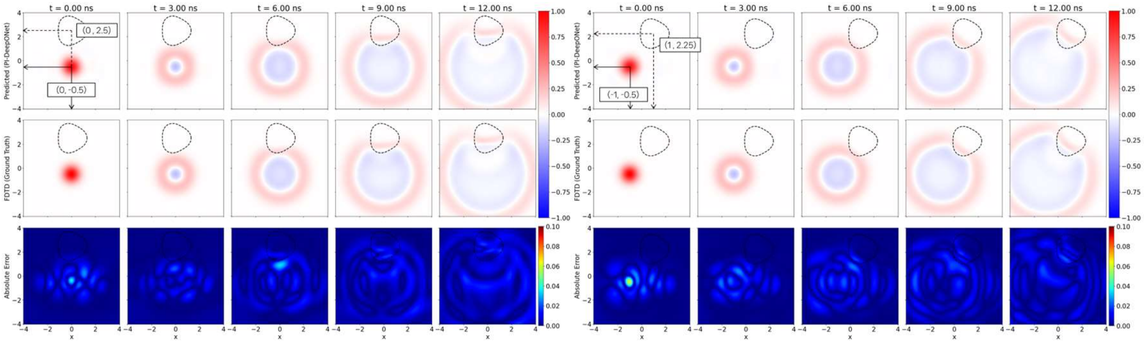

Figure. S8. Predicted field for irregularly shaped dielectric-inclusion/single-source configuration (ABC) in two different source and inclusion locations.

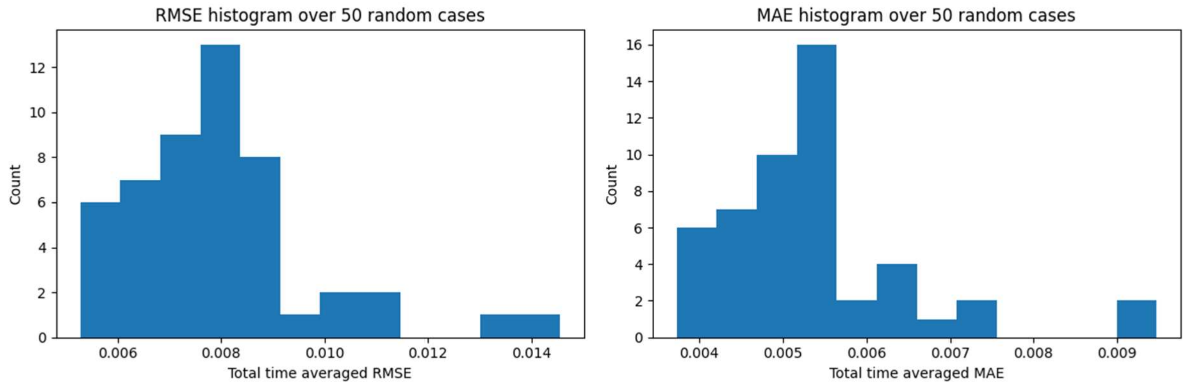

Figure. S9. Histograms of total time-averaged RMSE and MAE over 50 randomly sampled configurations (dielectric-inclusion/single source; Neumann BC).

Table S1. Comparison between interface-local and total RMSE under different boundary conditions.

		Time averaged RMSE		RMSE at t_{max}*	
		Interface-local	Total	Interface-local	Total
Dielectric-inclusion/ Single Source	Absorbing BC	8.48E-3	6.07E-3	1.29E-2	9.21E-3
	Reflecting BC	6.31E-3	5.63E-3	1.15E-2	8.09E-3
Multi-inclusion/ Single Source	Absorbing BC	8.67E-3	1.21E-2	1.64E-2	1.87E-2
	Reflecting BC	1.43E-2	1.031E-2	2.09E-2	1.913E-2

*t_{max} refers to t= 12ns.

Methods S1: Training code for Figure 6.

```
import os
import time
import datetime
import numpy as np
import tensorflow as tf
from tensorflow.keras.models import Model
from tensorflow.keras.layers import Dense
import matplotlib.pyplot as plt

from google.colab import drive
drive.mount('/content/drive')

class BranchNet(Model):
  def __init__(self, width= 2**6, p_dim= 2**6, depth= 20, name= "BranchNet"):
    super(BranchNet, self).__init__(name= name)
    self.hidden_layers= []
    for i in range(depth):
      layer_name= f'hidden_{i+1}'
      self.hidden_layers.append(Dense(width, activation='tanh', name= layer_name))
    self.output_layer= Dense(p_dim, activation= None, name= 'output_layer')
  def call(self, inputs):
    x= inputs
    for layer in self.hidden_layers:
      x= layer(x)
    return self.output_layer(x)

class TrunkNet(Model):
  def __init__(self, width= 2**6, p_dim= 2**6, depth= 20, name= "TrunkNet"):
    super(TrunkNet, self).__init__(name= name)
    self.hidden_layers= []
    for i in range(depth):
```

```
            layer_name= f'hidden_{i+1}'
            self.hidden_layers.append(Dense(width, activation='tanh', name= layer_name))
        self.output_layer= Dense(p_dim, activation= None, name= 'output_layer')
    def call(self, inputs):
        x= inputs
        for layer in self.hidden_layers:
            x= layer(x)
        return self.output_layer(x)

class PI_DeepONet(Model):
    def __init__(self, branch_net, trunk_net, name= "PI_DeepONet"):
        super(PI_DeepONet, self).__init__(name= name)
        self.branch_net= branch_net
        self.trunk_net= trunk_net
        self.b= tf.Variable(0.0, dtype=tf.float32, name='bias')

    def eps_fun(self, x, y, x1, y1, w= 4.0, h= 2.0):
        cond_x1= tf.logical_and(x>= x1- w/2, x<= x1+ w/2)
        cond_y1= tf.logical_and(y>= y1- h/2, y<= y1+ h/2)
        is_in_block1= tf.logical_and(cond_x1, cond_y1)
        epsilon_r= tf.where(is_in_block1, 4.0, 1.0)
        return epsilon_r

    def call(self, branch_inputs, t, x, y, x1, y1):
        trunk_inputs= tf.concat([t, x, y, x1, y1], axis= 1)
        branch_output= self.branch_net(branch_inputs)
        trunk_output= self.trunk_net(trunk_inputs)
        u_pred= tf.reduce_sum(branch_output * trunk_output, axis= 1, keepdims= True)
        return u_pred+ self.b

    def get_initial_condition(self, branch_inputs, t_ini, x_ini, y_ini, x1_ini, y1_ini):
        with tf.GradientTape() as tape:
            tape.watch(t_ini)
```

```
            u_ini= self.call(branch_inputs, t_ini, x_ini, y_ini, x1_ini, y1_ini)
        u_t_ini= tape.gradient(u_ini, t_ini)
        return u_ini, u_t_ini

    def get_boundary_derivatives(self, branch_bnd_x, t_bnd_x, x_bnd_x, y_bnd_x, x1_bnd_x, 
y1_bnd_x,
                                 branch_bnd_y, t_bnd_y, x_bnd_y, y_bnd_y, x1_bnd_y, 
y1_bnd_y):
        with tf.GradientTape(persistent=True) as tape:
            tape.watch([x_bnd_x, y_bnd_y])
            u_bnd_x= self.call(branch_bnd_x, t_bnd_x, x_bnd_x, y_bnd_x, x1_bnd_x, y1_bnd_x)
            u_bnd_y= self.call(branch_bnd_y, t_bnd_y, x_bnd_y, y_bnd_y, x1_bnd_y, y1_bnd_y)
        u_x_bnd= tape.gradient(u_bnd_x, x_bnd_x)
        u_y_bnd= tape.gradient(u_bnd_y, y_bnd_y)
        del tape
        return u_x_bnd, u_y_bnd

    def pde(self, branch_inputs, t, x, y, x1, y1):
        with tf.GradientTape(persistent=True) as tape:
            tape.watch(t)
            tape.watch(x)
            tape.watch(y)
            u= self.call(branch_inputs, t, x, y, x1, y1)
            u_t= tape.gradient(u, t)
            u_x= tape.gradient(u, x)
            u_y= tape.gradient(u, y)
        u_tt= tape.gradient(u_t, t)
        u_xx= tape.gradient(u_x, x)
        u_yy= tape.gradient(u_y, y)
        del tape

        epsilon_r_val= self.eps_fun(x, y, x1, y1)

        residual= u_tt- (1.0/ epsilon_r_val)* (u_xx+ u_yy)
```

```
    return residual

def prp_dat_deeponet(t, x, y, N_ini, N_bnd, N_pde,
                     x0_range=(-2., 2.), y0_range=(-1., 0.),
                     x1_range=(-2., 2.), y1_range=(2., 3.)):
  t_star, x_star, y_star= np.meshgrid(t, x, y)
  t_star, x_star, y_star= t_star.reshape(-1, 1), x_star.reshape(-1, 1), y_star.reshape(-1, 1)
  TX= np.c_[t_star, x_star, y_star]
  lb= tf.cast(tf.constant(tf.reduce_min(TX, axis= 0)), dtype=tf.float32)
  ub= tf.cast(tf.constant(tf.reduce_max(TX, axis= 0)), dtype=tf.float32)

  def create_branch_input(num_samples):
    x0= tf.random.uniform((num_samples, 1), x0_range[0], x0_range[1], dtype=tf.float32)
    y0= tf.random.uniform((num_samples, 1), y0_range[0], y0_range[1], dtype=tf.float32)
    return tf.concat([x0, y0], axis= 1)
  def create_block_location(num_samples):
    x1= tf.random.uniform((num_samples, 1), x1_range[0], x1_range[1], dtype=tf.float32)
    y1= tf.random.uniform((num_samples, 1), y1_range[0], y1_range[1], dtype=tf.float32)
    return tf.concat([x1, y1], axis= 1)

  # BranchNet Init
  branch_ini= create_branch_input(N_ini)
  x0_ini= branch_ini[:, 0:1]
  y0_ini= branch_ini[:, 1:2]
  # TrunkNet Init
  t_ini= tf.ones((N_ini, 1), dtype=tf.float32)* lb[0]
  x_ini= tf.random.uniform((N_ini, 1), lb[1], ub[1], dtype=tf.float32)
  y_ini= tf.random.uniform((N_ini, 1), lb[2], ub[2], dtype=tf.float32)
  block_loc_ini= create_block_location(N_ini)
  trunk_ini= tf.concat([t_ini, x_ini, y_ini, block_loc_ini], axis= 1)
  # u(x, y, 0)
  u_ini_true= tf.exp(-((x_ini- x0_ini)**2)/ 0.5)* tf.exp(-((y_ini- y0_ini)**2)/ 0.5)
  # BranchNet PDE
```

```
    branch_pde= create_branch_input(N_pde)
    # TrunkNet PDE
    t_pde= tf.random.uniform((N_pde, 1), lb[0], ub[0], dtype=tf.float32)
    x_pde= tf.random.uniform((N_pde, 1), lb[1], ub[1], dtype=tf.float32)
    y_pde= tf.random.uniform((N_pde, 1), lb[2], ub[2], dtype=tf.float32)
    block_loc_pde= create_block_location(N_pde)
    trunk_pde= tf.concat([t_pde, x_pde, y_pde, block_loc_pde], axis= 1)
    # BranchNet BND
    N_bnd_half= N_bnd//2
    branch_bnd= create_branch_input(N_bnd)
    block_loc_bnd= create_block_location(N_bnd)
    block_loc_bnd_x= block_loc_bnd[:N_bnd_half, :]
    block_loc_bnd_y= block_loc_bnd[N_bnd_half:, :]
    # TrunkNet BND_x
    t_bnd_x= tf.random.uniform((N_bnd_half, 1), lb[0], ub[0], dtype=tf.float32)
    x_bnd_x= lb[1]+ (ub[1]- lb[1])* tf.keras.backend.random_bernoulli((N_bnd_half, 1), 0.5,
dtype=tf.float32)
    y_bnd_x= tf.random.uniform((N_bnd_half, 1), lb[2], ub[2], dtype=tf.float32)
    trunk_bnd_x= tf.concat([t_bnd_x, x_bnd_x, y_bnd_x, block_loc_bnd_x], axis= 1)
    # TrunkNet BND_y
    t_bnd_y= tf.random.uniform((N_bnd_half, 1), lb[0], ub[0], dtype=tf.float32)
    x_bnd_y= tf.random.uniform((N_bnd_half, 1), lb[1], ub[1], dtype=tf.float32)
    y_bnd_y= lb[2]+ (ub[2]- lb[2])* tf.keras.backend.random_bernoulli((N_bnd_half, 1), 0.5,
dtype=tf.float32)
    trunk_bnd_y= tf.concat([t_bnd_y, x_bnd_y, y_bnd_y, block_loc_bnd_y], axis= 1)
    # Integrate
    data_batch= {
        'initial': (branch_ini, trunk_ini, u_ini_true),
        'pde': (branch_pde, trunk_pde),
        'boundary_x': (branch_bnd[:N_bnd_half, :], trunk_bnd_x),
        'boundary_y': (branch_bnd[N_bnd_half:, :], trunk_bnd_y)
    }
    return data_batch
```

```
@tf.function
def train_step(model, optimizer, w_ini, w_pde, w_bnd, data_batch):
    branch_ini, trunk_ini, u_ini_true= data_batch['initial']
    branch_pde, trunk_pde= data_batch['pde']
    branch_bnd_x, trunk_bnd_x= data_batch['boundary_x']
    branch_bnd_y, trunk_bnd_y= data_batch['boundary_y']

    with tf.GradientTape() as tape:
        u_pred_ini, u_t_ini= model.get_initial_condition(
                branch_ini, trunk_ini[:, 0:1], trunk_ini[:, 1:2], trunk_ini[:, 2:3],
                trunk_ini[:, 3:4], trunk_ini[:, 4:5])

        loss_u= tf.reduce_mean(tf.square(u_ini_true- u_pred_ini))
        loss_ut= tf.reduce_mean(tf.square(u_t_ini))
        loss_ini= loss_u+ loss_ut

        pde_residual= model.pde(
                branch_pde, trunk_pde[:, 0:1], trunk_pde[:, 1:2], trunk_pde[:, 2:3],
                trunk_pde[:, 3:4], trunk_pde[:, 4:5])
        loss_pde= tf.reduce_mean(tf.square(pde_residual))

        u_x_bnd, u_y_bnd= model.get_boundary_derivatives(
                branch_bnd_x, trunk_bnd_x[:, 0:1], trunk_bnd_x[:, 1:2], trunk_bnd_x[:, 2:3],
                trunk_bnd_x[:, 3:4], trunk_bnd_x[:, 4:5],
                branch_bnd_y, trunk_bnd_y[:, 0:1], trunk_bnd_y[:, 1:2], trunk_bnd_y[:, 2:3],
                trunk_bnd_y[:, 3:4], trunk_bnd_y[:, 4:5])
        loss_bnd= tf.reduce_mean(tf.square(u_x_bnd))+ tf.reduce_mean(tf.square(u_y_bnd))

        total_loss= w_ini* loss_ini+ w_pde* loss_pde+ w_bnd* loss_bnd

    grads= tape.gradient(total_loss, model.trainable_variables)
    optimizer.apply_gradients(zip(grads, model.trainable_variables))
```

```python
    return total_loss, loss_ini, loss_pde, loss_bnd

if __name__== "__main__":
    tmin, tmax, nt= 0., 4., 601
    xmin, xmax, nx= -4., 4., 81
    ymin, ymax, ny= -4., 4., 81
    t_= np.linspace(tmin, tmax, nt)
    x_= np.linspace(xmin, xmax, nx)
    y_= np.linspace(ymin, ymax, ny)

    n_epoch= 150000
    lr= 5e-5
    w_ini= 5.0
    w_pde= 2.5
    w_bnd= 1.0

    branch_net= BranchNet(width= 2**7, p_dim= 2**7, depth= 30)
    trunk_net= TrunkNet(width= 2**7, p_dim= 2**7, depth= 30)
    pideeponet_model= PI_DeepONet(branch_net, trunk_net)
    optimizer= tf.keras.optimizers.Adam(learning_rate= lr)

    history_epochs = []
    history_total_loss = []
    history_ic_loss = []
    history_pde_loss = []
    history_bnd_loss = []

    print("Learning Started")
    start_time= time.time()
    for epoch in range(n_epoch):
        data_batch= prp_dat_deeponet(t_, x_, y_, N_ini= int(3e4), N_bnd= int(1e4), N_pde= int(7e4),
                                    x0_range= (-2., 2.), y0_range= (-1., 0.),
                                    x1_range=(-2., 2.), y1_range=(2., 3.))
```

```
        total_loss, loss_ini, loss_pde, loss_bnd= train_step(pideeponet_model, optimizer, w_ini,
w_pde, w_bnd, data_batch)


        if epoch % 50 == 0:
            elapsed= time.time()- start_time
            print(f"Epoch {epoch}: Total Loss={total_loss:.4e}, IC Loss={loss_ini:.4e}, PDE
Loss={loss_pde:.4e}, BND Loss={loss_bnd:.4e}, Time: {elapsed:.2f}s")
            history_epochs.append(epoch)
            history_total_loss.append(total_loss.numpy())
            history_ic_loss.append(loss_ini.numpy())
            history_pde_loss.append(loss_pde.numpy())
            history_bnd_loss.append(loss_bnd.numpy())
            start_time= time.time()
    print("Learning Finished")


    plt.figure(figsize=(10, 6))
    plt.plot(history_epochs, history_total_loss, label='Total Loss')
    plt.plot(history_epochs, history_ic_loss, label='IC Loss')
    plt.plot(history_epochs, history_pde_loss, label='PDE Loss')
    plt.plot(history_epochs, history_bnd_loss, label='BND Loss')
    plt.xlabel('Epoch')
    plt.ylabel('Loss')
    plt.yscale('log')
    plt.legend()
    plt.grid(True, which="both", ls="--")
    plt.show()


    plt.figure(figsize=(10, 6))
    plt.plot(history_epochs, history_total_loss, label='Total Loss', color="black")
    plt.xlabel('Epoch')
    plt.ylabel('Loss')
    plt.yscale('log')
    plt.legend()
    plt.grid(True, which="both", ls="--")
```

```
plt.show()

print("Building model before saving weights...")
dummy_branch_input = tf.zeros((1, 2), dtype=tf.float32) # Branch: (x0, y0)
dummy_t = tf.zeros((1, 1), dtype=tf.float32)
dummy_x = tf.zeros((1, 1), dtype=tf.float32)
dummy_y = tf.zeros((1, 1), dtype=tf.float32)
dummy_x1= tf.zeros((1, 1), dtype=tf.float32)
dummy_y1= tf.zeros((1, 1), dtype=tf.float32)

_ =pideeponet_model(dummy_branch_input, dummy_t, dummy_x, dummy_y, dummy_x1,
dummy_y1)

save_path=
"/content/drive/MyDrive/PINN/Generalization2/5OneDielectricSingleSource_V1.weights.h5"
pideeponet_model.save_weights(save_path)
print("Model saved")
```

Methods S2: Inference code for Figure 6.

```
import os
import time
import datetime
import numpy as np
import tensorflow as tf
import matplotlib.pyplot as plt
from matplotlib.patches import Rectangle
from tensorflow.keras.models import Model
from tensorflow.keras.layers import Dense

from google.colab import drive
drive.mount('/content/drive')

class BranchNet(Model):
  def __init__(self, width= 2**6, p_dim= 2**6, depth= 10, name= "BranchNet"):
    super(BranchNet, self).__init__(name= name)
    self.hidden_layers= []
    for i in range(depth):
      layer_name= f'hidden_{i+1}'
      self.hidden_layers.append(Dense(width, activation='tanh', name= layer_name))
    self.output_layer= Dense(p_dim, activation= None, name= 'output_layer')
  def call(self, inputs):
    x= inputs
    for layer in self.hidden_layers:
      x= layer(x)
    return self.output_layer(x)

class TrunkNet(Model):
  def __init__(self, width= 2**6, p_dim= 2**6, depth= 10, name= "TrunkNet"):
    super(TrunkNet, self).__init__(name= name)
    self.hidden_layers= []
```

```
        for i in range(depth):
            layer_name= f'hidden_{i+1}'
            self.hidden_layers.append(Dense(width, activation='tanh', name= layer_name))
        self.output_layer= Dense(p_dim, activation= None, name= 'output_layer')
    def call(self, inputs):
        x= inputs
        for layer in self.hidden_layers:
            x= layer(x)
        return self.output_layer(x)

class PI_DeepONet(Model):
    def __init__(self, branch_net, trunk_net, name= "PI_DeepONet"):
        super(PI_DeepONet, self).__init__(name= name)
        self.branch_net= branch_net
        self.trunk_net= trunk_net
        self.b= tf.Variable(0.0, dtype=tf.float32, name='bias')

    @tf.function
    def call(self, branch_inputs, t, x, y, x1, y1):
        trunk_inputs= tf.concat([t, x, y, x1, y1], axis= 1)
        branch_output= self.branch_net(branch_inputs)
        trunk_output= self.trunk_net(trunk_inputs)
        u_pred= tf.reduce_sum(branch_output * trunk_output, axis= 1, keepdims= True)
        return u_pred+ self.b

def get_epsilon_map(x, y, block1_params):
    x1_min, x1_max= block1_params['center'][0]- block1_params['size'][0]/2, 
block1_params['center'][0]+ block1_params['size'][0]/2
    y1_min, y1_max= block1_params['center'][1]- block1_params['size'][1]/2, 
block1_params['center'][1]+ block1_params['size'][1]/2
    is_in_block1= np.logical_and(np.logical_and(x>= x1_min, x<= x1_max), np.logical_and(y>= 
y1_min, y<= y1_max))
    epsilon_r= np.ones_like(x, dtype=np.float32)
    epsilon_r= np.where(is_in_block1, block1_params['epsilon_r'], epsilon_r)
```

```
    return epsilon_r

def FDTD(nt, nx, ny, dt, dx, dy, c_real, BC, initial_u_map, epsilon_r_map):
    u= np.zeros((nt, nx, ny), dtype= np.float32)
    u[0, :, :]= initial_u_map
    u[1, :, :]= u[0, :, :]
    c_map= c_real/ np.sqrt(epsilon_r_map)

    t0= time.time()
    for n in range(1, nt- 1):
        if n% int(100)== 0:
            print(f">>>>> FDM computing... n: {n}, nt: {nt}")

        for i in range(1, nx- 1):
            for j in range(1, ny- 1):
                c_ij= c_map[i, j]
                u[n+ 1, i, j]= 2* u[n, i, j] - u[n- 1, i, j] \
                                + (c_ij* dt/ dx)**2* (u[n, i+ 1, j] - 2* u[n, i, j] + u[n, i- 1, j]) \
                                + (c_ij* dt/ dy)**2* (u[n, i, j+ 1] - 2* u[n, i, j] + u[n, i, j- 1])
        if BC== "Neu":
            for i in range(1, nx- 1):
                u[n+ 1, i, 0]= u[n+1, i, 1]
                u[n+ 1, i, -1]= u[n+ 1, i, -2]
            for j in range(1, ny- 1):
                u[n+ 1, 0, j]= u[n+ 1, 1, j]
                u[n+ 1, -1, j]= u[n+ 1, -2, j]
    t1= time.time()
    print(f"FDTD Completed--{t1- t0: .2f}s")
    return u

def main():
    width, p_dim, depth= 2**7, 2**7, 30
```

```
BC= "Neu"
c_real= 3e8

tmin, tmax, nt= 0., 4., 601
xmin, xmax, nx= -4., 4., 481
ymin, ymax, ny= -4., 4., 481

t_space= np.linspace(tmin, tmax, nt)
x_space= np.linspace(xmin, xmax, nx)
y_space= np.linspace(ymin, ymax, ny)

dt= t_space[1] - t_space[0]
dx= x_space[1] - x_space[0]
dy= y_space[1] - y_space[0]

t_real_space= t_space/ c_real
dt_real= t_real_space[1]- t_real_space[0]

X, Y= np.meshgrid(x_space, y_space)
X_flat= X.flatten()[:, np.newaxis].astype(np.float32)
Y_flat= Y.flatten()[:, np.newaxis].astype(np.float32)

branch_net_inference= BranchNet(width= width, p_dim= p_dim, depth= depth)
trunk_net_inference= TrunkNet(width= width, p_dim= p_dim, depth= depth)
inference_model= PI_DeepONet(branch_net_inference, trunk_net_inference)

load_path=
"/content/drive/MyDrive/PINN/Generalization2/5OneDielectricSingleSource_V2.weights.h5"

dummy_t= tf.zeros((1, 1))
dummy_x= tf.zeros((1, 1))
dummy_y= tf.zeros((1, 1))
dummy_x1= tf.zeros((1, 1))
```

```
dummy_y1= tf.zeros((1, 1))
dummy_branch= tf.zeros((1, 2))
inference_model(dummy_branch, dummy_t, dummy_x, dummy_y, dummy_x1, dummy_y1)
inference_model.load_weights(load_path)
print("Model loaded successfully.")

# x1_range=(-2., 2.), y1_range=(2., 3.)
block1_params_infer = {'center': [-0, 2.5], 'size': [4.0, 2.0], 'epsilon_r': 4.0}

# x0_range= (-2, 2), y0_range= (-1, 0)
x0_new, y0_new= -1.5, -0.5
branch_input_new= tf.constant([[x0_new, y0_new]], dtype=tf.float32)

print("Inference Started")
u_pred_all= np.zeros((nt, nx, ny))
start_time= time.time()

x1_infer= block1_params_infer['center'][0]
y1_infer= block1_params_infer['center'][1]
X1_flat= np.ones_like(X_flat)* x1_infer
Y1_flat= np.ones_like(Y_flat)* y1_infer
for i, t_val in enumerate(t_space):
    T_flat= np.ones_like(X_flat)* t_val
    u_hat= inference_model(branch_input_new, T_flat, X_flat, Y_flat, X1_flat, Y1_flat)
    u_pred_all[i, :, :]= tf.reshape(u_hat, (nx, ny)).numpy()
end_time= time.time()
print(f"Inference Completed--{end_time- start_time: .2f}s")

print("FDTD Started")
initial_map_FDTD= np.exp(-((X- x0_new)**2)/ 0.5)* np.exp(-((Y- y0_new)**2)/ 0.5)
epsilon_r_map_2d= get_epsilon_map(X, Y, block1_params_infer)
u_FDTD= FDTD(nt, nx, ny, dt_real, dx, dy, c_real, BC, initial_map_FDTD, epsilon_r_map_2d)
```

```
abs_error= np.abs(u_pred_all- u_FDTD)
squared_error= (u_pred_all- u_FDTD)**2

mae= np.mean(abs_error)
mse= np.mean(squared_error)
rmse= np.sqrt(mse)

print(f"\nMean Absolute Error: {mae:.4e}")
print(f"Root Mean Squared Error: {rmse:.4e}")

v_min, v_max= -1, 1
print(f"Min/Max of PINN prediction: {np.min(u_pred_all)}, {np.max(u_pred_all)}")

block_center= block1_params_infer['center']
block_size= block1_params_infer['size']
bottom_left_corner= [block_center[0]- block_size[0]/2, block_center[1]- block_size[1]/2]

probe_points= [(0, -2), (0, 2)]

fig, axes= plt.subplots(1, len(probe_points), figsize=(8* len(probe_points), 4))
if len(probe_points)== 1:
    axes= [axes]
for ax, (px, py) in zip(axes, probe_points):
    idx_x= np.argmin(np.abs(x_space- px))
    idx_y= np.argmin(np.abs(y_space- py))

    pinn_timeseries= u_pred_all[:, idx_y, idx_x]
    fdtd_timeseries= u_FDTD[:, idx_y, idx_x]

    ax.plot(t_real_space* 1e9, fdtd_timeseries, label= 'FDTD', linestyle= '--', linewidth= 3, color=
'red')
    ax.plot(t_real_space* 1e9, pinn_timeseries, label= 'PI-DeepONet', linewidth= 3, color= 'black')
    ax.set_title(f"Ez(t) at (x={x_space[idx_x]:.1f}, y={y_space[idx_y]:.1f})", fontsize=16)
```

```python
    ax.set_xlabel("Time [ns]", fontsize=16)
    ax.set_ylabel("Ez", fontsize=16)
    ax.set_xlim(0, 12)
    ax.tick_params(axis='both', which='major', labelsize=14)
    ax.legend(fontsize=16)
    ax.grid(True)
plt.tight_layout()
plt.show()

times_to_plot_ns = np.array([0., 3., 6., 9., 12.])
all_times_ns = t_real_space * 1e9
indices_to_plot = [np.argmin(np.abs(all_times_ns - t_ns)) for t_ns in times_to_plot_ns]
actual_times_ns = [all_times_ns[i] for i in indices_to_plot]

num_rows = 3
num_cols = len(indices_to_plot) # 5

plot_data = [u_pred_all, u_FDTD, abs_error]
plot_cmaps = ['bwr', 'bwr', 'gray']
plot_vmins = [v_min, v_min, 0]
plot_vmaxs = [v_max, v_max, 0.1]
row_titles = ["Predicted (PI-DeepONet)", "FDTD (Ground Truth)", "Absolute Error"]

fig, axes = plt.subplots(num_rows, num_cols,
                         figsize=(25, 15),
                         constrained_layout=True)

pcm_objects= [[None for _ in range(num_cols)] for _ in range(num_rows)]

for i in range(num_rows): # 0: Predicted, 1: FDTD, 2: Error
    for j in range(num_cols): # 0: 0ns, 1: 3ns, ...
        ax = axes[i, j]
        t_idx = indices_to_plot[j]
```

```
        data = plot_data[i][t_idx, :, :]
        cmap = plot_cmaps[i]
        vmin = plot_vmins[i]
        vmax = plot_vmaxs[i]
        pcm = ax.pcolormesh(X, Y, data, shading='auto', cmap=cmap, vmin=vmin, vmax=vmax)
        pcm_objects[i][j] = pcm

        if i == 0:
            ax.set_title(f"t = {actual_times_ns[j]:.2f} ns", fontsize=25)

        if j == 0:
            ax.set_ylabel(row_titles[i], fontsize=20)
        else:
            ax.set_yticklabels([])

        if i == num_rows - 1:
            ax.set_xlabel("x", fontsize=20)
        else:
            ax.set_xticklabels([])

        ax.tick_params(axis='both', which='major', labelsize=20)
        ax.set_aspect('equal', 'box')

        rect = Rectangle(bottom_left_corner, block_size[0], block_size[1],
                         edgecolor='black', facecolor='none', linewidth= 2, linestyle= '--')
        ax.add_patch(rect)

cbar1 = fig.colorbar(pcm_objects[0][-1], ax=axes[0:2, -1],
                     shrink=1, pad=0.01)
cbar1.ax.tick_params(labelsize=15)
cbar2 = fig.colorbar(pcm_objects[2][-1], ax=axes[2, -1],
                     shrink=1, pad=0.01)
cbar2.ax.tick_params(labelsize=15)
```

```
    plt.show()

if __name__== "__main__":
    main()
```

Methods S3: Training code for Figure 7.

Here, everything is same except for ‘class PI_DeepONet’ and ‘def train_step’.

```
class PI_DeepONet(Model):
  def __init__(self, branch_net, trunk_net, c= 1.0, name= "PI_DeepONet"):
    super(PI_DeepONet, self).__init__(name= name)
    self.branch_net= branch_net
    self.trunk_net= trunk_net
    self.b= tf.Variable(0.0, dtype=tf.float32, name='bias')
    self.c= tf.constant(c, dtype=tf.float32)

  def eps_fun(self, x, y, x1, y1, w= 4.0, h= 2.0):
    cond_x1= tf.logical_and(x>= x1- w/2, x<= x1+ w/2)
    cond_y1= tf.logical_and(y>= y1- h/2, y<= y1+ h/2)
    is_in_block1= tf.logical_and(cond_x1, cond_y1)
    epsilon_r= tf.where(is_in_block1, 4.0, 1.0)
    return epsilon_r

  def call(self, branch_inputs, t, x, y, x1, y1):
    trunk_inputs= tf.concat([t, x, y, x1, y1], axis= 1)
    branch_output= self.branch_net(branch_inputs)
    trunk_output= self.trunk_net(trunk_inputs)
    u_pred= tf.reduce_sum(branch_output * trunk_output, axis= 1, keepdims= True)
    return u_pred+ self.b

  def get_initial_condition(self, branch_inputs, t_ini, x_ini, y_ini, x1_ini, y1_ini):
    with tf.GradientTape() as tape:
      tape.watch(t_ini)
      u_ini= self.call(branch_inputs, t_ini, x_ini, y_ini, x1_ini, y1_ini)
    u_t_ini= tape.gradient(u_ini, t_ini)
    return u_ini, u_t_ini
```

```
    def get_boundary_gradients(self, branch_inputs, t, x, y, x1, y1):
        with tf.GradientTape(persistent=True) as tape:
            tape.watch(t)
            tape.watch(x)
            tape.watch(y)
            u= self.call(branch_inputs, t, x, y, x1, y1)
        u_t= tape.gradient(u, t)
        u_x= tape.gradient(u, x)
        u_y= tape.gradient(u, y)
        del tape
        return u, u_t, u_x, u_y

    def pde(self, branch_inputs, t, x, y, x1, y1):
        with tf.GradientTape(persistent=True) as tape:
            tape.watch(t)
            tape.watch(x)
            tape.watch(y)
            u= self.call(branch_inputs, t, x, y, x1, y1)
            u_t= tape.gradient(u, t)
            u_x= tape.gradient(u, x)
            u_y= tape.gradient(u, y)
        u_tt= tape.gradient(u_t, t)
        u_xx= tape.gradient(u_x, x)
        u_yy= tape.gradient(u_y, y)
        del tape

        epsilon_r_val= self.eps_fun(x, y, x1, y1)

        residual= u_tt- (1.0/ epsilon_r_val)* (u_xx+ u_yy)
        return residual

@tf.function
def train_step(model, optimizer, w_ini, w_pde, w_bnd, data_batch):
```

```
branch_ini, trunk_ini, u_ini_true= data_batch['initial']
branch_pde, trunk_pde= data_batch['pde']
branch_bnd_x, trunk_bnd_x= data_batch['boundary_x']
branch_bnd_y, trunk_bnd_y= data_batch['boundary_y']

lb= data_batch['lb']
ub= data_batch['ub']

with tf.GradientTape() as tape:
    u_pred_ini, u_t_ini= model.get_initial_condition(
            branch_ini, trunk_ini[:, 0:1], trunk_ini[:, 1:2], trunk_ini[:, 2:3],
            trunk_ini[:, 3:4], trunk_ini[:, 4:5])

    loss_u= tf.reduce_mean(tf.square(u_ini_true- u_pred_ini))
    loss_ut= tf.reduce_mean(tf.square(u_t_ini))
    loss_ini= loss_u+ loss_ut

    pde_residual= model.pde(
            branch_pde, trunk_pde[:, 0:1], trunk_pde[:, 1:2], trunk_pde[:, 2:3],
            trunk_pde[:, 3:4], trunk_pde[:, 4:5])
    loss_pde= tf.reduce_mean(tf.square(pde_residual))

    t_bnd_x= trunk_bnd_x[:, 0:1]
    x_bnd_x= trunk_bnd_x[:, 1:2]
    y_bnd_x= trunk_bnd_x[:, 2:3]
    x1_bnd_x= trunk_bnd_x[:, 3:4]
    y1_bnd_x= trunk_bnd_x[:, 4:5]
    _, u_t_bnd_x, u_x_bnd, _= model.get_boundary_gradients(
            branch_bnd_x, t_bnd_x, x_bnd_x, y_bnd_x, x1_bnd_x, y1_bnd_x)

    epsilon_r_bnd_x = model.eps_fun(x_bnd_x, y_bnd_x, x1_bnd_x, y1_bnd_x)
    v_bnd_x = 1.0 / tf.sqrt(epsilon_r_bnd_x)
```

```
    sign_x= tf.where(tf.equal(x_bnd_x, lb[1]), -1.0, 1.0)
    res_bnd_x= u_t_bnd_x+ v_bnd_x* sign_x* u_x_bnd
    loss_bnd_x= tf.reduce_mean(tf.square(res_bnd_x))

    t_bnd_y= trunk_bnd_y[:, 0:1]
    x_bnd_y= trunk_bnd_y[:, 1:2]
    y_bnd_y= trunk_bnd_y[:, 2:3]
    x1_bnd_y= trunk_bnd_y[:, 3:4]
    y1_bnd_y= trunk_bnd_y[:, 4:5]
    _, u_t_bnd_y, _, u_y_bnd= model.get_boundary_gradients(
        branch_bnd_y, t_bnd_y, x_bnd_y, y_bnd_y, x1_bnd_y, y1_bnd_y)

    epsilon_r_bnd_y = model.eps_fun(x_bnd_y, y_bnd_y, x1_bnd_y, y1_bnd_y)
    v_bnd_y = 1.0 / tf.sqrt(epsilon_r_bnd_y)

    sign_y= tf.where(tf.equal(y_bnd_y, lb[2]), -1.0, 1.0)
    res_bnd_y= u_t_bnd_y+ v_bnd_y* sign_y* u_y_bnd
    loss_bnd_y= tf.reduce_mean(tf.square(res_bnd_y))

    loss_bnd= loss_bnd_x+ loss_bnd_y

    total_loss= w_ini* loss_ini+ w_pde* loss_pde+ w_bnd* loss_bnd

grads= tape.gradient(total_loss, model.trainable_variables)
optimizer.apply_gradients(zip(grads, model.trainable_variables))

return total_loss, loss_ini, loss_pde, loss_bnd
```

Methods S4: Inference code for Figure 7.

For the inference, the same code for reflecting BC would work if you change 'BC= "Neu"' in 'def main()' into 'BC= "ABC"'.
